# Complex Growth of Benzamide Form I: Effect of Additives, Solution Flow, and Surface Rugosity

**DOI:** 10.1021/acs.cgd.2c00842

**Published:** 2022-09-27

**Authors:** Caroline A. Offiler, Cláudio P. Fonte, Weronika Kras, Petros Neoptolemou, Roger J. Davey, Thomas Vetter, Aurora J. Cruz-Cabeza

**Affiliations:** † Department of Chemical Engineering, University of Manchester, Manchester M13 9PL, U.K.; ‡ Department of Solid Form Science, H. Lundbeck A/S, Ottiliavej 9, 2500 Copenhagen, Denmark

## Abstract

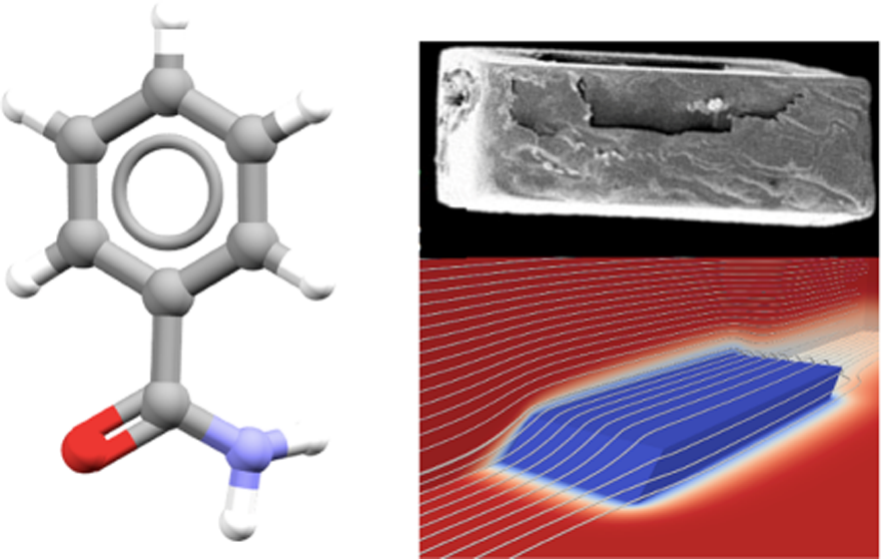

Understanding crystal growth kinetics is of great importance for the development and manufacturing of crystalline molecular materials. In this work, the impact of additives on the growth kinetics of benzamide form I (BZM-I) crystals has been studied. Using our newly developed crystal growth setup for the measurement of facet-specific crystal growth rates under flow, BZM-I growth rates were measured in the presence of various additives previously reported to induce morphological changes. The additives did not have a significant impact on the growth rates of BZM-I at low concentrations. By comparison to other systems, these additives could not be described as “effective” since BZM-I showed a high tolerance of the additives’ presence during growth, which may be a consequence of the type of growth mechanisms at play. Growth of pure BZM-I was found to be extremely defected, and perhaps those defects allow the accommodation of impurities. An alternative explanation is that at low additive concentrations, solid solutions are formed, which was indeed confirmed for a few of the additives. Additionally, the growth of BZM-I was found to be significantly affected by solution dynamics. Changes in some facet growth rates were observed with changes in the orientation of the BZM-I single crystals relative to the solution flow. Of the two sets of facets involved in the growth of the width and length of the crystal, the {10*l̅*} facets were found to be greatly affected by the solution flow while the {011} facets were not affected at all. Computational fluid dynamics simulations showed that solute concentration has higher gradients at the edges of the leading edge {10*l̅*} facets, which can explain the appearance of satellite crystals. {10*l̅*} facets were found to show significant structural rugosity at the molecular level, which may play a role in their mechanism of growth. The work highlights the complexities of measuring crystal growth data of even simple systems such as BZM-I, specifically addressing the effect of additives and fluid dynamics.

## Abstract 2

This work highlights the complexities of measuring crystal growth data of even simple systems such as benzamide form I, specifically addressing the effect of additives and fluid dynamics on crystal growth kinetics. A link is found between structural rugosity and growth kinetics controlled by mass transfer.

## 1 Introduction

Many factors can affect crystal morphologies during crystallization including supersaturation, solvent type, temperature, or the presence of small amounts of other substances—either wanted (additives) or unwanted (impurities). Crystal morphology is an important feature of materials as it can influence downstream processing as well as the properties of the final products.^1−4^ Dramatic morphological changes can result from the addition of trace amounts of an additive to the crystallization environment. By altering the nature and amount of additives used in a crystallization experiment, a desired change in crystal morphologies may be achieved.^5^ Typically, an effective additive displays a level of structural similarity to the solute molecule together with a molecular variation that enables insertion of the additive in a specific crystal surface (through its similarity) as well as disruption of subsequent growth (through its variation).^6^ The uptake of the additive onto available crystal facets will differ depending on the local stereochemistry and potential intermolecular interactions.^7^ Additives can be tailored to adsorb selectively onto certain facets (but not others) and achieve the desired crystal shape.^8^

**Figure 1:**
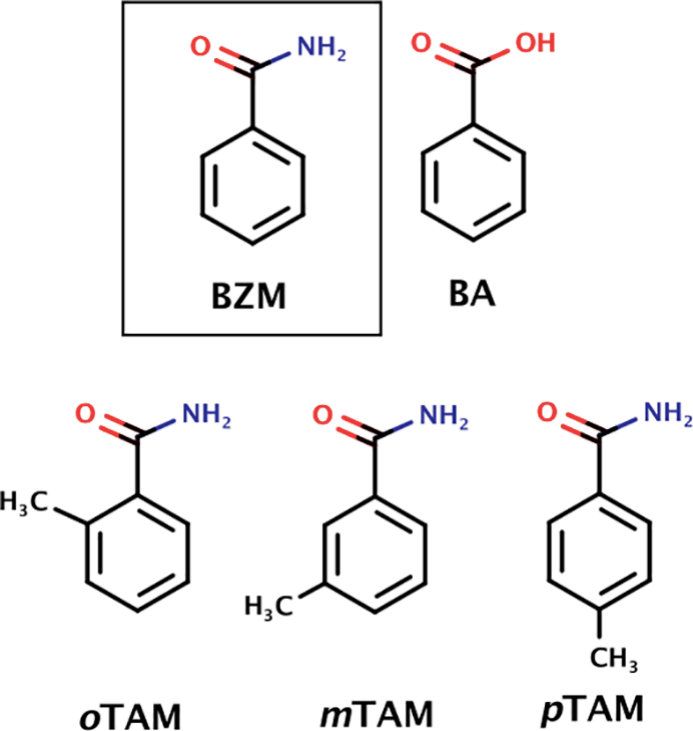
Molecular structures of benzamide (BZM) and the additives used in this work including benzoic acid (BA), *o*-toluamide (*o*TAM), *m*-toluamide (*m*TAM), and *p*-toluamide (*p*TAM).

One of the first studies to demonstrate these effects appeared in the 1982 work of Berkovitch-Yellin et al.,^9^ who reported the facet-specific influence of additives on the crystal morphology of benzamide form I (BZM-I). They studied the effects of a number of additives including benzoic acid (BA), *o*-toluamide (*o*TAM), and *p*-toluamide (*p*TAM) on the crystal morphology of BZM-I (Figure 1). Each additive yielded a morphological change, which was rationalized from a knowledge of the additive molecular structure together with the known molecular packing of BZM-I on the relevant crystal surfaces.^9^ BA was shown to inhibit growth along the *b*-axis by insertion onto the {011} faces disrupting the hydrogen-bonded ribbon of amide dimers. *o*TAM was shown to incorporate onto the {10*l̅*} facets through hydrogen bonding with its bulky methyl group disrupting the “regular deposition of substrate molecules”. Similarly, *p*TAM was shown to incorporate onto the {020} face resulting in thinner BZM-I platelets.

In a more recent study also on BZM-I, Blagden et al.^10^ demonstrated the effect of 2′-aminoacetophenone (APP) on its growth and suggested that, rather than acting as a tailor-made additive, APP acted as a motif capper where it hydrogen bonded to the exposed amine and carbonyl groups at the end of the dimer ribbons, preventing their further growth. They observed that rather than inhibiting *b*-axis growth in the presence of increasing amounts of APP (0.1–10%), significant crystal aggregation occurred. Aggregated crystals were overlapping along the *c*-axis, with new crystals rotated by 90° relative to the original. This orientation was attributed to the bonding of the APP to the BZM-I in both aggregated crystals. An inhibition of the *c*-axis growth was also seen with this additive.

The conclusions of both these relevant studies on the growth of BZM-I in the presence of additives were drawn based on the observed morphological changes using slow cooling crystallization methods. No actual measurements of the influence of these additives on growth rates were performed, though of course the morphological outcome is indeed a result of the changes in the growth rates. In this context, the present work seeks to utilize crystal growth rate data along with morphological observations to understand further the growth behavior of the historically important BZM-I system. Here, our newly developed automated method was used to study the facet-specific growth rates^11^ of BZM-I pure as well as in the presence of the additives shown in Figure 1. The technique combines a crystal growth flow cell with image processing allowing for efficient retrieval of facet-specific growth rates. There are many advantages to this setup including: (i) the use of the constant flow of solution (thus, there is no supersaturation depletion during growth), (ii) growth rate data are derived for each individual facet, and (iii) data collection and analyses are optimized compared to previous designs.^12,13^

## 2 Experimental Section

### 2.1 Materials

All compounds were used as received without further purification. The molecular structures of additives used in this work are shown in Figure 1. Benzamide (form I, 99% purity), benzoic acid (≥99.5% purity), and 2′-aminoacetophenone (≥98% purity) were purchased from Sigma-Aldrich. *o*-Toluamide (99% purity) was purchased from FluoroChem, and *m*-toluamide (99% purity) and *p*-toluamide (>98% purity) from Alfa Aesar. The solvent isopropanol (IPA, ≥99.5% purity) was purchased from Honeywell.

### 2.2 Powder X-ray Diffraction

The Bruker D2-Phaser diffractometer was used for the powder X-ray diffraction (PXRD) characterization of crystalline powders. Diffractograms were collected using Cu Kα radiation (λ = 1.54 Å) in the 2θ range between 5 and 40° (step size 0.02°). PXRD was used to confirm that all benzamide samples corresponded to the BZM-I polymorph.

### 2.3 Scanning Electron Microscopy

Scanning electron microscopy (SEM) was performed using a Quanta 200 microscope operated under vacuum. Prior to imaging, selected crystals were carefully transferred onto a carbon conductive tape on top of an aluminum stub and were coated with approximately 10–13 nm of platinum using a Cressington 108 auto Sputter Coater.

### 2.4 Differential Scanning Calorimetry

Differential scanning calorimetry (DSC) was conducted using a TA Instruments DSC 2500. Approximately 3–5 mg of dried slurry crystallites were loaded onto Tzero aluminum pans, and the pans were sealed with aluminum lids. Samples were heated from room temperature until after melting at 5 °C min^–1^.

### 2.5 Solubility of BZM-I in IPA and in the Presence of Additives

Solubility values for BZM-I in pure IPA and in IPA in the presence of various amounts of additives (BA, *o*TAM, *p*TAM, and *m*TAM) were measured at 15 °C (±0.2 °C) using the gravimetric method.

### 2.6 Supersaturation

Supersaturation in this manuscript is defined as the ratio of the mol fractions as defined in eq 1 where *x*_BZM_^ss^ is the mol fraction of BZM in the supersaturated solution and *x*_BZM_^equiv^ is the solubility at the appropriate additive content.
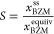
1

### 2.7 Slow Cooling Crystallizations

Slow cooling crystallizations were performed to gain insights into the impact of the additives on the overall crystal morphology of BZM-I. Solutions were prepared by stirring BZM in IPA at 30 °C in the presence of the required amounts of additives for 1 h until all solids had dissolved, after which the stirrer bar was removed. Solutions were then cooled from 30 to 15 °C at 1 °C per hour, at which point supersaturations of approximately 1.25 were generated. The solutions were kept at 15 °C until crystals of approximately 400 μm were produced (as judged by eye, typically obtained after up to 1 day), after which crystals were isolated through vacuum filtration.

### 2.8 Production of Seed Crystals

Seed crystals of BZM-I were prepared by slow evaporation from BZM solutions in IPA/water mixtures (90:10 vol %) as this afforded thicker single crystals which were easier to handle. Solutions were prepared by stirring 5 g of BZM in 30 mL of the solvent mixture at approximately 60 °C (using a hot plate and a magnetic stirrer) until all solids had dissolved. Solutions were then cooled to room temperature and left to evaporate in Petri dishes covered in pierced parafilm. Once crystals of approximately 400 μm in size were formed, they were isolated by vacuum filtration. Crystals to be used as seeds in growth experiments were first examined under cross polarizers with an Axioplan2 microscope (Zeiss, Jena) to identify good-quality single crystals of high transparency and lack of visible defects. Crystals of the appropriate size for the growth cell (700–2000 μm) were used as prepared, while larger crystals were cut to a suitable size using a knife.

### 2.9 Growth Rate Measurements

Growth rate experiments on BZM-I in both pure solutions and in the presence of additives were carried out in a growth cell under flow conditions (flow cell, FC). The FC setup and image analysis algorithms used in data extraction have been described in detail elsewhere.^11^ Briefly, at its core sits a small quartz flow cell through which solution at a controlled concentration can flow. The cell is immersed in a water bath set to 15 ± 0.01 °C. For this work, the cell was connected to a solution reservoir whose flow, controlled by a pump connected to a flow meter, was set to 35 g/h, which equates to a flow velocity in the flow cell of approximately 0.62 mms^–1^ and a Reynolds number of 0.7 (assuming the properties of the solution are the same as that of pure IPA), the ratio between the magnitude of inertial and viscous forces in the flow—indicating steady laminar flow around a seed crystal placed in the cell. The design of the setup with clear Perspex windows on the water bath allows for imaging, thanks to a light-emitting diode (LED) light source placed below and a camera positioned above the flow cell (one image is recorded every 10 s).

Additive levels used in these experiments varied slightly according to their individual behavior. For example, in the case of BA, growth rates were measured in the presence of 1 and 5 mol % to compare with previous work.^9,10^ On the other hand, in the case of the toluamides (*o*TAM, *m*TAM, *p*TAM), an additive level of 0.5 mol % was used since higher levels resulted in unwanted nucleation of additional crystals in the cell. Before starting the experiments, the seed crystals were partially dissolved in the growth cell to remove any defects. In experiments involving changes in crystal orientation, a needle was used to rotate the crystals. To prevent nucleation in the cell upon insertion of the needle, the temperature of the cell was temporarily increased before each reorientation operation.

A second growth cell was used to measure the growth rate of BZM-I under static conditions (static cell, SC). The SC setup consists of a sealed quartz cell (0.7 mL volume) in which the required solution and seed is introduced manually for each system measurement. As with the flow cell, the quartz cell is placed inside a water bath with clear Perspex windows to allow for imaging. The water bath is placed under an inverted microscope (Olympus CKX41), and images of the growing crystal are taken every 10 s. This setup has also been reported in detail elsewhere.^14^

Since all single crystals remain static during the measurements, crystal size information as a function of time is retrieved from the images using our in-house image analysis code.^10^ Due to BZM-I growing satellite crystals (see results), a small adjustment was made to the image analysis code so that a growth rate could be derived for each of the facet fronts and an average growth rate calculated with its standard derivation.

### 2.10 Crystal Thickness Characterization

Using the growth cell, the growth of facets along the crystal length and width were measured but not the thickness. Here, the effect of additives on the thickness of the BZM-I crystals, *c*-axis, under controlled conditions was also examined. Experiments were performed for BZM-I in the following conditions: pure, 10 mol % *m*TAM, 10 mol % *p*TAM, 0.5 mol % *m*TAM, and 0.5 mol % *p*TAM. For this, solutions of benzamide (and additive, if using) in IPA were prepared in jacketed vessels and stirred at 30 °C until all solids had dissolved. The stirrer bar was then removed, and the temperature was set to 15 °C at conditions that afford solutions with an initial supersaturation of 1.05. After 15 min, one seed crystal was placed in each vessel and was then left for at least 24 h. After this time, nucleation occurs, and the supersaturation can be assumed to be zero. Crystals were then isolated by vacuum filtration and placed on a transparent sample holder plate. Photo images were recorded with the aid of a camera attached to a telecentric lens. An image analysis algorithm was then used to identify single crystals and measure the size of each crystal in the populations.^15^ Recorded populations included: (a) a population of 31 crystals of BZM-I grown from pure IPA, (b) a population of 21 BZM-I grown from IPA in the presence of 0.5% *p*TAM, (c) a population of 40 BZM-I grown from IPA in the presence of 0.5% *m*TAM, (d) a population of 27 BZM-I grown from IPA in the presence of 10% *p*TAM, and (e) a population of 26 BZM-I grown from IPA in the presence of 10% *m*TAM.

The thickness of all crystals was measured using a chromatic confocal sensor IFS2405-3 supplied from Micro-Epsilon Messtechnik, which was integrated within our previously described imaging setup. Since the spotlight diameter of our chromatic confocal sensor is 9 μm, only particles that have a width (smallest *x*–*y* dimension) equal to 20 μm or larger were imaged. Each crystal was scanned once across the y-dimension to get its surface profile in a line of points. Data were acquired and processed with Matlab 2021a. Errors (spikes) in the sensor’s signal were removed using the movmedian function, and the white noise was removed using the moving average smoothing function. The method has been tested and calibrated relative to laser measurements. The confocal sensor will be explained in detail elsewhere (to be published), and the referenced paper gives details on the overall setup.^15^

### 2.11 Computational Fluid Dynamics Calculations

The flow field and concentration distribution inside the flow cell were computed by numerical solution of the Navier–Stokes equations for a fluid of density ρ and viscosity μ. Details of this are given in the ESI. The governing equations for the flow and mass transport were solved with the open-source Finite Volume CFD code OpenFOAM v9.^16^ Steady-state solutions of the flow and concentration fields were obtained with the solver simpleFoam using second-order interpolation schemes for the advective terms of the governing equations. The SIMPLE method was used to solve the velocity and pressure fields iteratively. The simulations were considered converged when the residuals of all of the equations reached values smaller than 10^–6^ during the iterative solution process.

The CFD simulations used a number of parameters representative of the growth experiments. This included a crystal size and aspect ratio typical of a BZM-I morphology (*L*_a_ = 1773 μm, *L*_b_ = 1484 μm, *L*_c_ = 164 μm) with an adopted 44.45° right rhombic prism crystal geometry representative of the angle between the {1̅0*l*} and the {002} families of planes. For the fluid, a density of 789 kg m^–3^ and a viscosity of 2.78 mPas were used and, for the solute, a diffusion coefficient of 1.1 × 10^–9^ m^2^ s^–1^. The flow and mass transport conditions were set for values of Reynolds and Péclet numbers equal to Re = 0.03 and Pe = 92, respectively, which correspond to typical values in the experiments. Simulations for two different crystal orientations were performed as detailed later. Moreover, it was assumed that the crystal growth mechanism is controlled by diffusive and advective mass transport phenomena as per the experimental observations reported below (see the Supporting Information). In that sense, a fast consumption rate of BZM solute was assumed from the inlet solution when in contact with any of the faces of the crystal. Further details on the governing equations and boundary conditions of the mathematical model and different aspects of its numerical solution are presented in the SI.

## 3 Face Indexing, Morphologies, and Defects

### 3.1 Crystal Structure and Face Indexing of BZM-I

**Figure 2:**
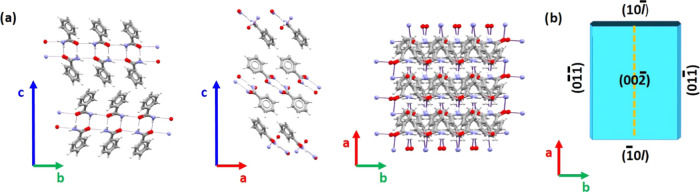
(a) Molecular packing view of BZM-I along the *a*-, *b*-, and *c*-axes. (b) Depiction of a face-indexed BZM-I morphology (BZAMID05 refcode).

The earliest single crystal determination of BZM-I available in the Cambridge Structural Database (CSD) dates back to 1959 (Penfold et al. monoclinic space group *P*2̅_1_c space group with *a* = 5.59 Å, *b* = 5.01 Å, *c* = 21.93 Å and β = 90.75°, CSD refcode BZAMID^17^). Many redeterminations of BZM-I now exist in the CSD, and here BZAMID05^18^ (with the lowest *R*-factor) is used throughout this manuscript. BZM forms hydrogen-bonded (HB) dimers, which further interact through HB side chains as well as a number of aromatic interactions. The continuous interactions along the three axis are: (a) aromatic stacking related by translation along the *a*-axis, (b) HB side chains with contributions from aromatic stacking along the *b*-axis, and (c) a combination of the strong HB dimers (not continuous) with weaker aromatic CH t-type ring to ring interactions along the *c*-axis (Figure 2a).

Early work by Leiserowitz et al.^9^ reported detailed face-indexed morphologies for BZM-I from several solvents. The plate-like habit is depicted in Figure 2b with faces indexed according to previous assignments. Clearly, the dominant facet in BZM-I plates is (002̅), thus implying that the *c*-axis is the slowest growing direction. This corresponds to the crystal thickness (*L*_c_). To reconfirm this assignment on our BZM-I samples, unground plates of crystallized BZM-I (see Figure 5 for images of typical crystals grown from IPA solution) were aligned on a PXRD sample holder, and a diffractogram was recorded. The dominance of the {002̅} facets in the plate morphology was confirmed, the data showing distinct preferential orientation with diffraction intensities due to the {00*l*} family of planes significantly dominating the pattern (see the SI).

Identification of the other two crystal directions on our plate morphologies, the *a*- and *b*-axes, was less straightforward and was done via microscopy making use of the required crystal point group symmetry, 2/m. To be consistent with the crystallography, the crystal morphology must show a mirror plane (dashed yellow line, Figure 2b) perpendicular to the *b*-axis so that facets perpendicular to the *b*-axis must have mirror symmetry. In the case of BZM-I, those facets correspond to the {011} family of planes (four planes in total). By contrast, the planes perpendicular to the *a*-axis, the (10*l̅*) and (1̅0*l*), are related by 2-fold symmetry. The exact value of the *l* index for the (10*l̅*) plane has been previously reported to vary with solvent and growth conditions and to lie between 1 and 4.^9,10,19^ The angle between the dominant (002̅) plane and the (10*l̅*) plane then increases from 105, 116, 127, and 135° as the *l* indices changes from 1̅ to 4̅, respectively. Thus, the higher the *l* index, the more clearly visible the (10*l̅*) plane becomes when the crystal is viewed down the *c*-axis (i.e., crystal sitting on its major, (002̅) face).

**Figure 3:**
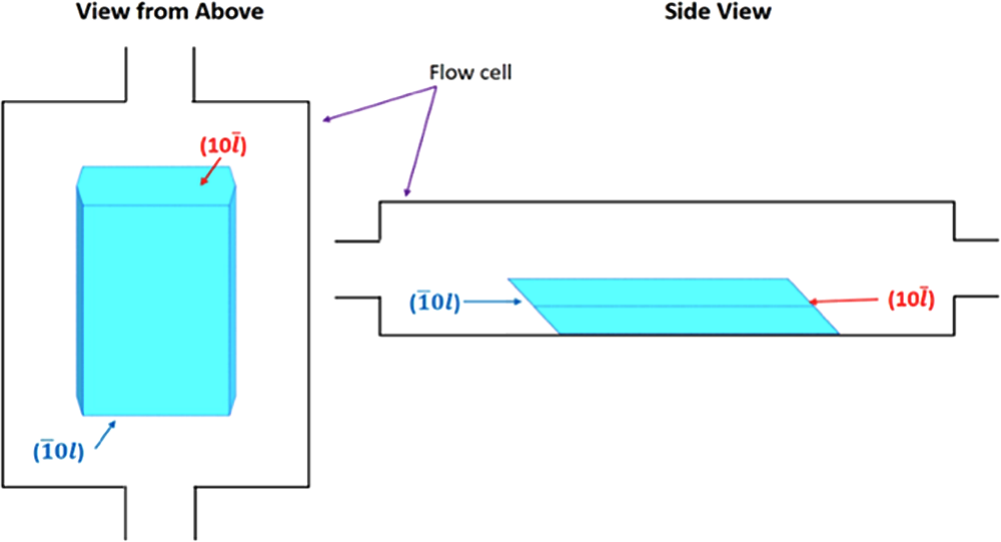
Geometry of BZM-I morphology and its orientation relative to the growth cell. The following naming convention is used for the {10*l̅*} faces: (10*l̅*) for faces looking upward (acute angle with regards to the sample holder plane) and (1̅0*l*) for faces looking downward (obtuse angle with regards to the sample holder plane).

Figure 3 provides a schematic diagram of a typical crystal sitting in the growth cell and viewed down the *c*-axis (left) and down the *b*-axis (right). It illustrates how the inherent crystal shape and symmetry (with the 2-fold axis aligned with the *b*-axis) results in only one of the {10*l̅*} facets (the (10*l̅*) in Figure 3) facing slightly upward toward the imaging camera, which is positioned above the cell. The slightly upward facing (10*l̅*) facet, however, may not be clearly visible if *l* is a low index. For clarity and consistency, when referring to the {10*l̅*} family of planes, the face tilted toward the imaging camera is referred to as the (10*l̅*) face (acute face relative to the sample holder, Figure 3), while the face tilted toward the sample holder is referred to as the (1̅0*l*) face (obtuse face relative to the sample holder, Figure 3); this is, of course, an arbitrary choice but a helpful distinction. When the distinction of upward and downward tilted facets in the {10*l̅*} family is not possible visually, the two different faces are referred to as either {10*l̅*}-*a* or {10*l̅*}-*b* arbitrarily.

**Figure 4:**
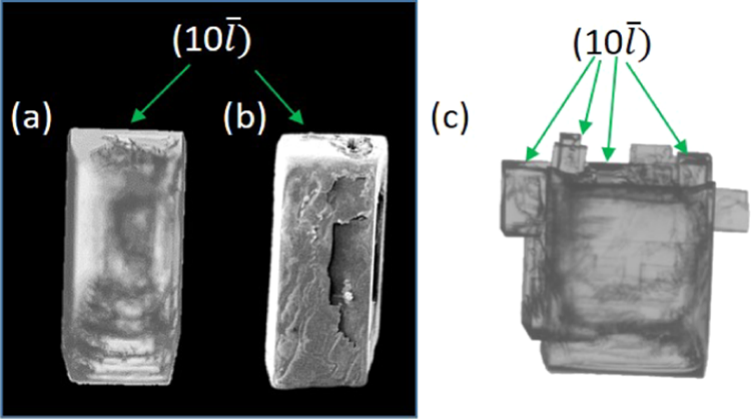
(a) Camera image of a BZM-I seed after growth in the FC for 1 h. (b) SEM image of the crystal shown in panel (a). (c) Crystal grown in the FC showing a satellite grown onto the (10*l̅*) facet.

The consequence of these morphological issues is that in growth experiments, the identification of the *a*-axis and the *b*-axis must be done for each crystal by imaging and identifying the required symmetry (as explained above and illustrated in Figure 2b). A typical indexing assignment based on images is shown in Figure 4a. Here, the (10*l̅*) facet of a BZM-I crystal growing in the flow cell (FC) has been identified based on the seed geometry and symmetry. To reconfirm such an assignment, an SEM image of the same crystal is shown in Figure 3b, where the different edges and facets can be seen even more clearly. This careful identification of the facets and axis is important since variations in morphology with solvent and additive mean that the long dimension of the plate does not necessarily correspond to the *a*-axis. *L_a_*, *L_b_*, and *L_c_* are used to refer to the dimension of the crystal along the *a*-, *b*- (length and or width), and *c*-axes (thickness), respectively. One final feature of the BZM-I morphology that is worth noting is that during growth, many cases were observed in which the crystal facets became unstable with the appearance of satellite crystals protruding from the seed crystal surface on one of the (10*l̅*) facets as seen in Figure 4c. This is discussed further in later sections.

### 3.2 Impact of Additives on BZM-I Solubility in IPA

The recorded solubility values of BZM-I in pure IPA and in the presence of additives at 15 °C are given in Table 1. At low additive levels (∼1 mol % or less), the overall changes to solubility are negligible (within the error of the method). However, at higher additive content, changes do become more apparent. For BA and *m*TAM, at 10 mol %, there is practically no change in the solubility. For both *o*TAM and *p*TAM, there is a considerable increase of solubility at 10 mol % from 0.037 pure to 0.056 and 0.045, respectively.

**Table 1:** Measured Solubility Values (in mol fraction) of BZM in IPA at 15 °C and in the Presence of Various Additives

impurity	impurity content	solubility (*x*_bzm_^equiv^)	impact of additive on solubility
none	none	0.037 ± 0.001	pure BZM-I
BA	0.5 mol % BA	0.037 ± 0.001	no change
	10 mol % BA	0.039 ± 0.001	
*o*TAM	0.5 mol % *o*TAM	0.043 ± 0.001	increase in solubility
	10 mol % *o*TAM	0.056 ± 0.001	
*m*TAM	0.5 mol % *m*TAM	0.037 ± 0.001	no change
	10 mol % *m*TAM	0.037 ± 0.001	
*p*TAM	0.5 mol % *p*TAM	0.037 ± 0.001	increase in solubility
	10 mol % *p*TAM	0.045 ± 0.001	

### 3.3 Impact of the Additives on the BZM-I Crystal Morphologies

**Figure 5:**
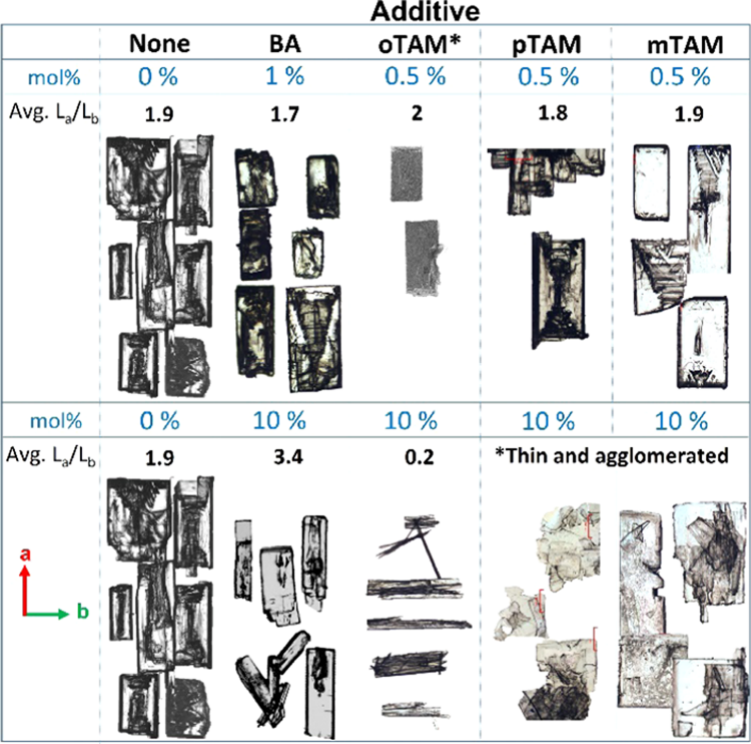
Morphological impact of additives at lower (0–0.5 mol %, upper part) and higher (10 mol %, lower part) concentrations on the morphologies of BZM-I grown from IPA by slow cooling. Crystals have been carefully aligned according to their *a*- and *b*-axes orientations. Due to agglomeration, the aspect ratio *L*_a_/*L*_b_ of a number of systems could not be determined. All images are optical except for *o*TAM at low concentration, which are SEM micrographs.

To reconfirm the overall impact of the additives on the resulting BZM-I morphologies reported in previous work, slow cooling crystallization experiments of BZM-I from IPA were performed.^5,10^ Experiments were done at a supersaturation of 1.25 since it gave a good balance between a reasonable crystallization time without resulting in agglomeration. Preliminary testing of supersaturation conditions, however, showed that supersaturation did not seem to have a big impact on the overall resulting morphologies and aspect ratio of BZM-I. The images in Figure 4 summarize our results. Here, the crystals have been manually aligned with their *a*-axes pointing upward. Overall, these morphologies are in good agreement with previously reported observations of Leiserowitz et al.^9,10^ At low additive contents, the morphological changes are small and hardly visible (Figure 5, upper part). At 10 mol % additive, however, the effects become very apparent (Figure 5, lower part). In agreement with previous work, *o*TAM inhibits the *a*-axis growth and BA the *b*-axis growth, while *p*TAM inhibits the *c*-axis growth. It was also found in this work that *m*TAM inhibits *c*-axis growth. Additives inhibiting *c*-axis growth result in very thin plates, which break easily and sit on top of each other.

### 3.4 Imaging of Defects on BZM-I Single Crystals

**Figure 6:**
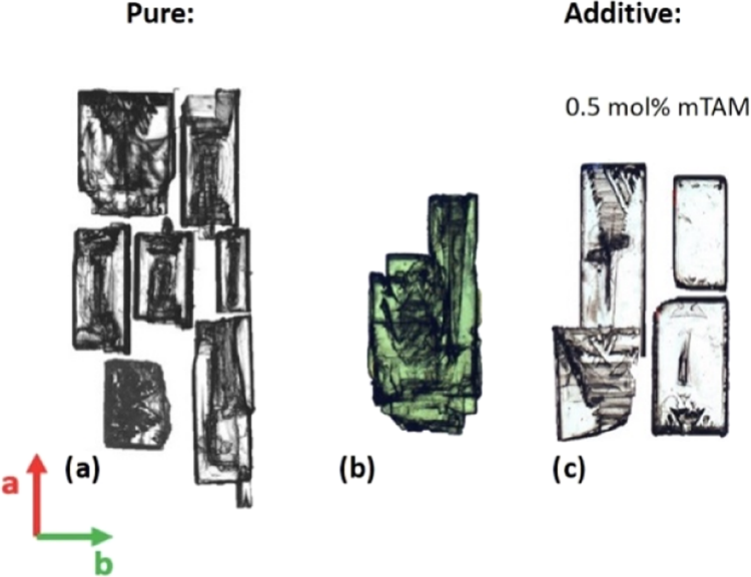
Micrographs of BZM-I crystals grown from slow cooling in IPA: (a) crystals grown from pure solution, (b) crystals grown from pure solution with many defects seen under polarized light, and (c) crystals grown in the presence of 0.5 mol % of *m*TAM.

**Figure 7:**
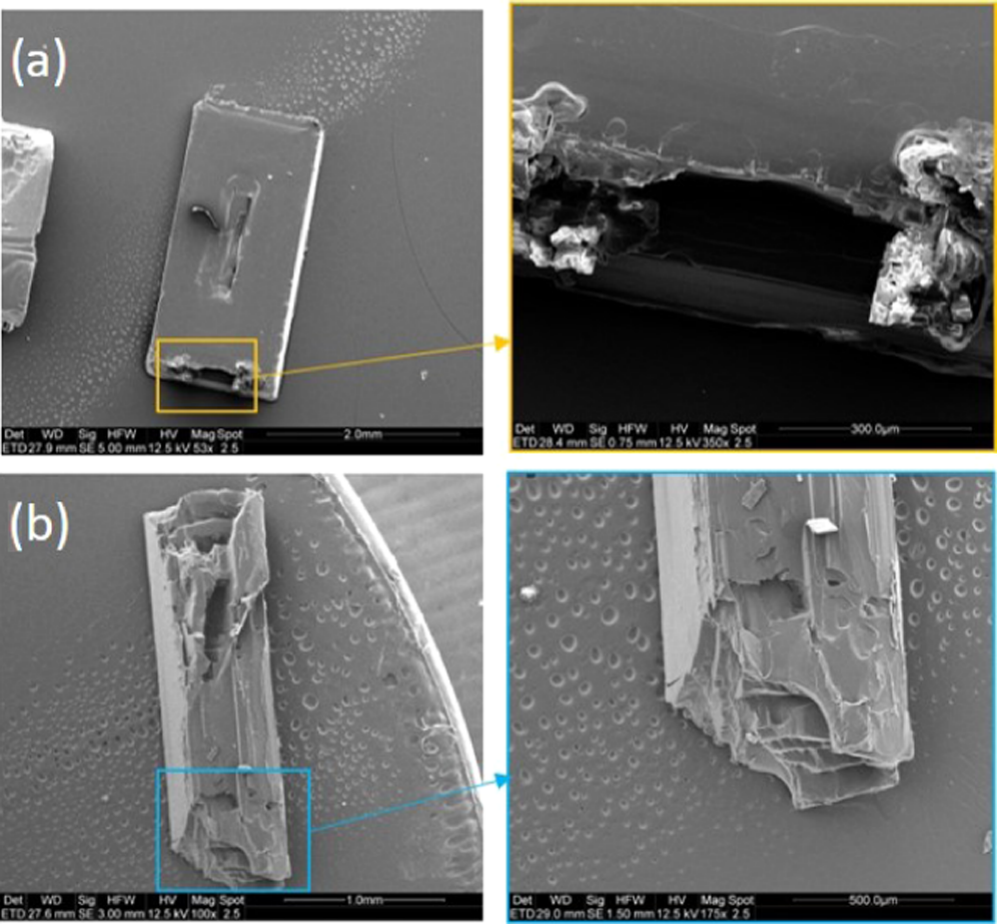
SEM images, with magnifications of specific regions, of BZM-I showing defects in crystals grown from IPA in the presence of 0.5 mol % *m*TAM (a) and 0.5% *p*TAM (b).

Images of single crystals of BZM-I with normal and polarized optical microscopy (Figure 6) and SEM (Figure 6) reveal many growth features such as liquid inclusions and nonuniform growth. For example, in Figure 6a, the crystals grown from pure solution show dark hourglass shadows resulting from inclusions in the {10*l̅*} growth sectors. In Figure 6c, similar effects are seen in the presence of *m*TAM, although in general it appears that the additives improve the optical quality of the crystals, with the inclusions being less apparent. In pure solution, the formation of these inclusions is also associated with irregular growth along the *a*-axis, resulting in single crystals which look like aggregates, as seen in Figure 5b. The origin of the inclusions suggests local supersaturation differences across a facet, highest at the edges and lower in the center leading to edges growing faster and trapping solvent at the center.^20^ This is confirmed by the SEM images in Figure 7a,b, which clearly show the formation of holes in the face centers. The implications of these observations are discussed in more detail in later sections.

## 4 Growth Rate Data

### 4.1 Seed Crystals

Seed crystals grown for rate measurements also had many defects. Attempts were made to improve the crystal quality, but neither a change in solvent (ethanol, IPA, and mixtures 90:10 vol % solvent/water mixtures were tested) nor recrystallization of BZM-I reduced the defective nature of the seeds produced. This suggests that the generation of inclusions, layers, and protuberances is the result of the growth mechanisms and not the growth environment. Seeds added to the growth cell were partially dissolved to remove perturbations before any growth experiments were started.

**Figure 8:**
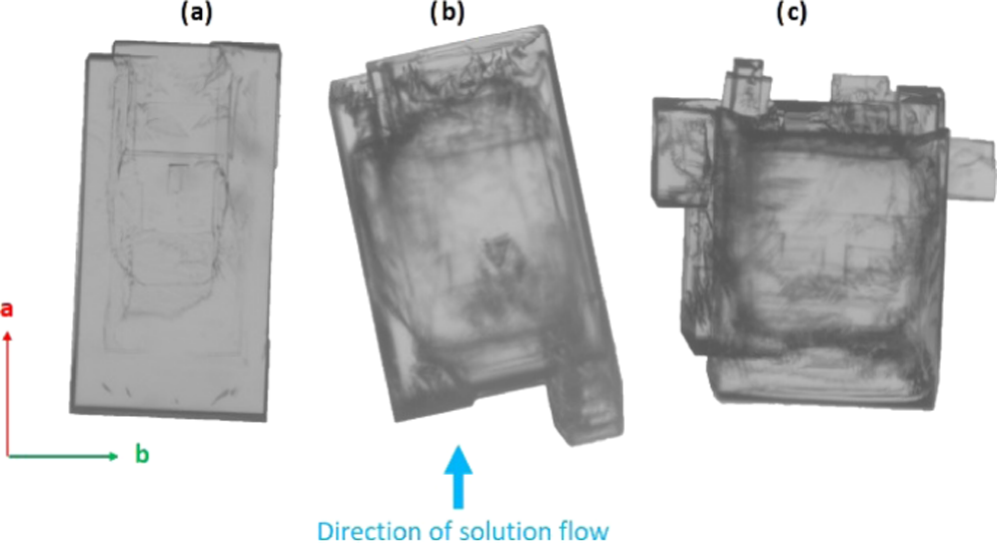
Image of pure BZM-I crystals after growth in the flow cell showing no protuberances after 21 mins of growth at *S* = 1.21 (a), protuberances along the *a*-axis after 1 h of growth at *S* = 1.35 (b), and protuberances along both *a*- and *b*-axes after 1 h of growth at *S* = 1.24 (c).

**Figure 9:**
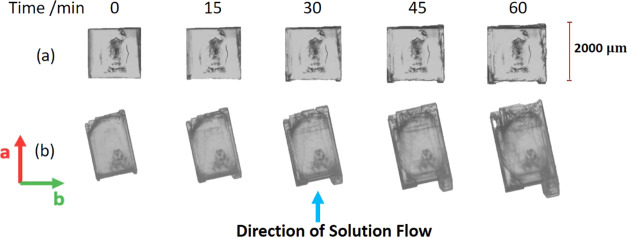
Images taken during the growth of a BZM-I seed crystal in the flow cell at *S* = 1.06 (a) and 1.35 (b). During the experiments, satellites of BZM-I appear on the {10*l̅*} facets.

As far as the growth of BZM-I crystal seeds in the cell was concerned, a range of behaviors was typically observed, varying from regular growth in which the overall morphology is preserved (Figure 8a) to irregular growth in which satellite crystals appear along the *a*-axis (Figure 8b) or along the *a*- and *b*-axes (Figure 8c). The figure shows two examples of a BZM-I seed grown in the flow cell (FC) at two supersaturations, 1.06 and 1.35, both of which lead to the appearance of satellite crystals on the {10*l̅*} facets throughout the experiment. These perturbations appear to originate from the corners of the {10*l̅*} faces (Figures 8 and 9). In Figure 9b, the top (1̅0*l*) facet is seen to comprise an array of satellites at 30 min; however, these satellites appear to grow together, and at 40 min, the surface reflattens. In Figure 9b, the bottom (10*l̅*) facet does not reflatten as the large satellite is growing over twice as fast as the corresponding (10*l̅*) facet on the seed crystal. It is noted that the most regular growth occurs at the lowest supersaturations. In these measurements with protuberances, the growth rate of each facet front was determined (major front as well as satellites) and an average growth rate was calculated. Finally, for the experiments done under flow, unless otherwise indicated, the crystals were oriented so that the solution flow was parallel to the *a*-axis and perpendicular to the *b*-axis as far as it was practically possible. The reason for this is explained in subsequent sections. In most experiments, crystals were not oriented with the flow with regards to the two geometries of the {10*l̅*} facets. This was because crystals were introduced into the growth cell in undersaturated conditions to prevent unwanted nucleation, this reduced the size of the (10*l̅*) face making identification difficult via the camera images until after the crystal had grown a sufficient amount.

### 4.2 Impact of Solution Flow and Crystal Orientation on the Growth of BZM-I

**Figure 10:**
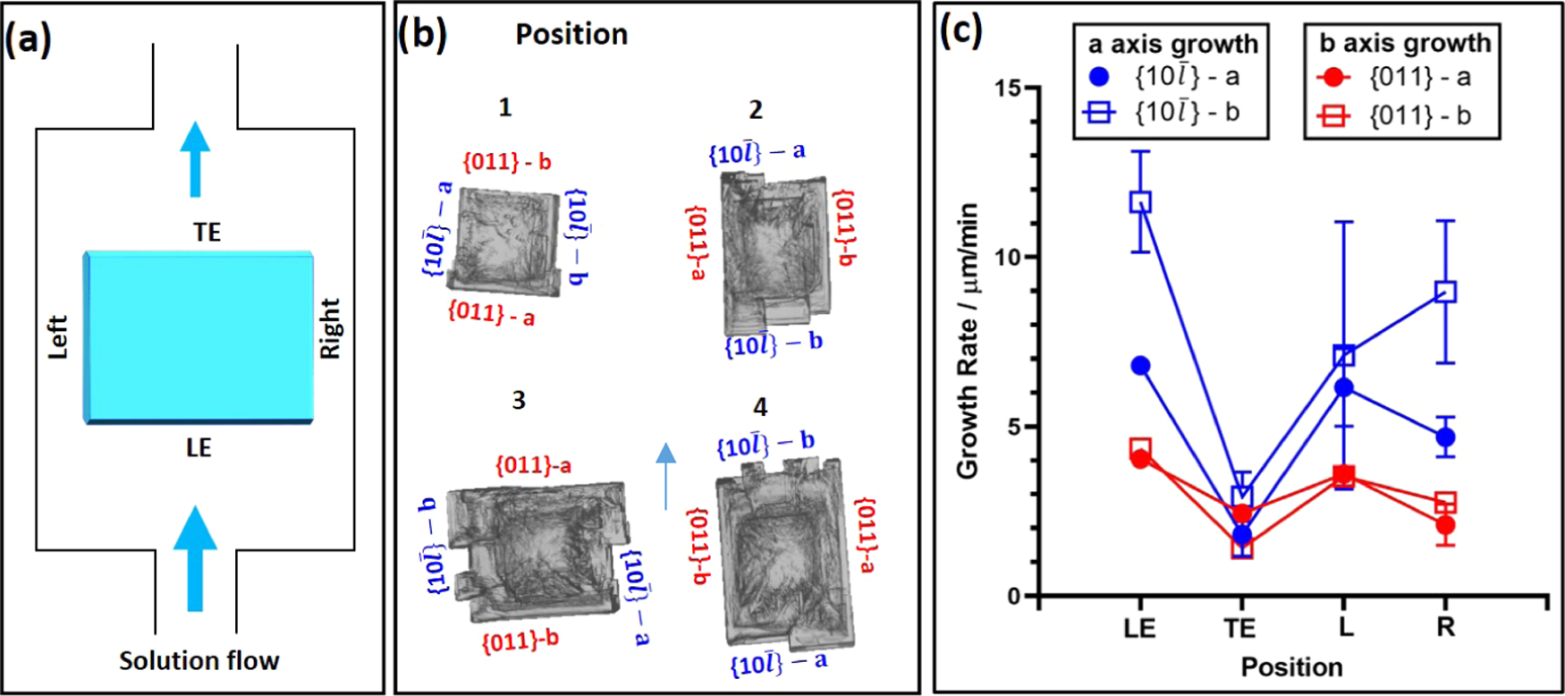
(a) Orientation of the facets of the growing crystal in the cell relative to the solution flow, (b) images of a BZM-I reoriented crystal, and (c) growth rate of each facet in each of the four positions. Growth from IPA at 15 °C with a *S* = 1.25.

To test the impact of solution flow on the growth of BZM-I crystals, growth rates were measured in pure solution in the flow cell at *S* = 1.25. The growing crystal was rotated 90° every half an hour so that rates were measured with each face lying either perpendicular to the solution flow (leading or trailing edges) or parallel to the solution flow (side edges). The solution velocity was kept constant throughout the experiment. The results indicated a definite influence of solution flow on the growth rates of some facets, with Figure 10 showing the effect of changing crystal orientation on the rates. As seen schematically in Figure 10a, the four crystal orientations for each facet relative to the solution flow are defined as: leading edge (LE, directed into the flow), trailing edge (TE, face shielded from the flow), and left and right (parallel to the flow, side faces). Figure 10b then provides images from one experiment with the crystal in each of its four unique positions. Incidentally, these images also show how the {10*l̅*} surfaces develop “satellite” crystals while the {011} facets maintain their faceted shape. Each satellite grows at a different rate; for example, in position 2 (Figure 10b), three distinct growth fronts can be seen on the {10*l̅*}-*b* facet with growth rates of 13.7, 10.2, and 11.0 μm min^–1^ from the left to right. For the purpose of analysis, the overall growth rate of the facet is taken as the average of all three satellites, giving a growth rate of 11.6 μm min^–1^. From these data (Figure 10c), it can be seen that while the {011} facets grow at rates between ∼ 2 and 4 μm min^–1^ irrespective of their orientation, the {10*l̅*} facets grow at different rates depending on their orientation relative to the solution flow (Figure 10c).

In the LE orientations, the {10*l̅*}-*b* facet grows significantly faster than the {10*l̅*}-*a* facet (12 vs 6 μm min^–1^). However, in the TE orientation, the growth of the {10*l̅*}-*b* and {10*l̅*}-*a* facets are essentially identical and significantly slower than in the LE orientations (growth rates falling to just 2.9 μm min^–1^ in line with the rates of the {011} facets). After careful image analysis, the {10*l̅*}-*b* facet was identified as the (1̅0*l*) geometry having the obtuse angle with the sample holder, while the {10*l̅*}-*a* facet was identified as the (10*l̅*) geometry having the acute angle with the sample holder thus this face being tilted upward toward the top camera. The differences in the experimental growth rates of these two face orientations of {10*l̅*} faces is evident. The differences, however, are believed to be due to either growth rate dispersion or differences in impurity profiles on these two facets rather than their specific orientations relative to the sample holder having an impact on the mass transfer of solute.^21,22^ This will be explored further with fluid dynamics simulations in a later section.

**Figure 11:**
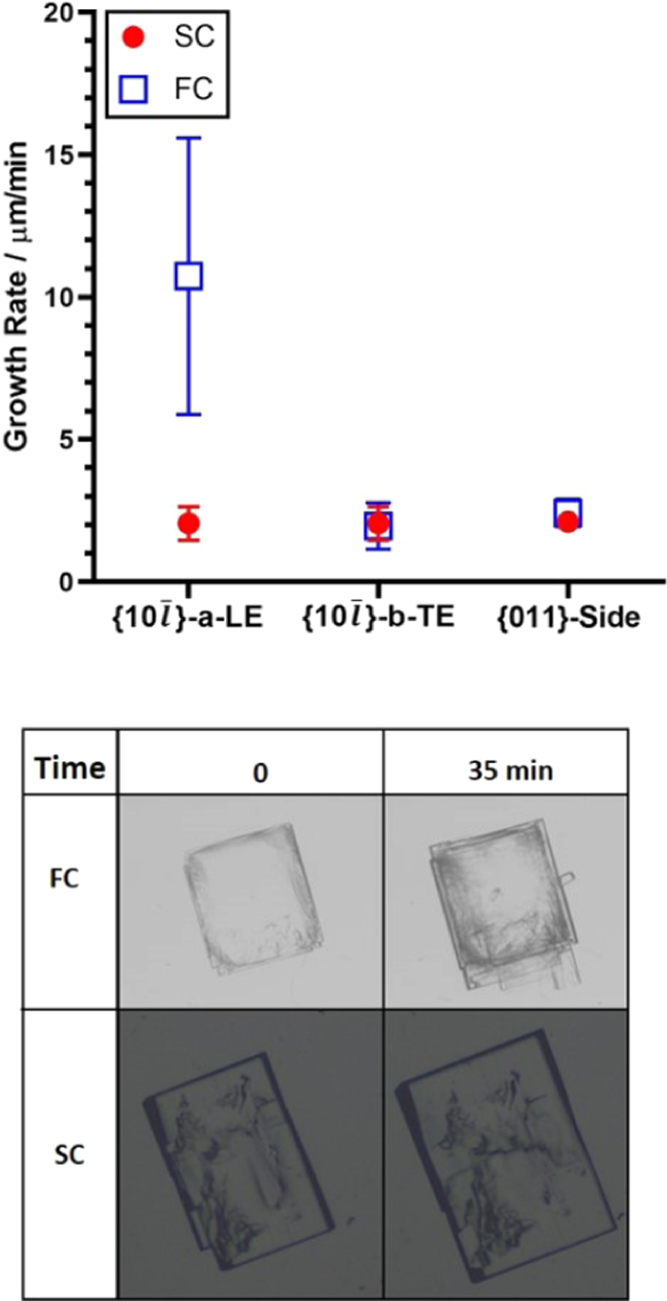
Comparison of crystal growth rates for a pure BZM-I crystal derived from the static growth cell (SC) and the flow growth cell (FC). Crystals grown at 15 °C and *S* = 1.25.

These results were confirmed by performing further experiments whereby two crystals were grown under the same growth conditions, one with solution flow (grown in the flow cell, FC) and the other one under no solution flow (grown in the static cell, SC). In the FC, the crystal is orientated with the {10*l̅*} facets normal to the solution flow, one at the LE and one at the TE. Figure 11 compares the derived growth rates of both crystals. The FC satellites are seen on the {10*l̅*}-*a*-LE facet, and the overall growth rate is an average of these, with the standard deviation given as the error bar. The {011} facets grow at very similar rates in both growth cells, confirming little to no impact on the solution flow. As far as the {10*l̅*} facets are concerned, the dependence on solution flow is again evident: the {10*l̅*} facets in the TE-flow position grow at an equivalent rate to the {10*l̅*} facets in the SC, while {10*l̅*} facet in the LE position has a rate which is over an order of magnitude higher than in the SC. All of these experiments reconfirm the finding that the {10*l̅*} facets exhibit increased growth rates when directed into the solution flow. Finally, the crystal grown in the SC has a more equant and better-faceted shape than the crystal grown in the FC (Figure 11); in fact, in the SC, the crystal became better facetted as it grew while single crystals grown under flow often contained many protuberances. Further confirmation of the impact of solution flow was observed in an experiment in which two BZM-I crystals were grown in the FC, both at a supersaturation of 1.35 but with differing solution flow rates: (i) at 5 g h^–1^ (approx. 0.088 mm s^–1^) and (ii) at 35 g h^–1^ (approx. 0.62 mm s^–1^)—the results are summarized in Table 2. The {10*l̅*}-*a*-LE facet grows faster under higher flow rates while the {10*l̅*}-*b*-TE facet is less affected (with some variations due to different seeds used as well as the errors of the method). All of these data suggest that growth kinetics of the {10*l̅*} facet is at least partially mass transport controlled and affected by both the flow rate of solution as well as the orientation of the facet relative to the flow. All data shown in the next sections of this paper are obtained at a flow rate of 35 g h^–1^ (approx. 0.62 mm s^–1^).

**Table 2:** Comparison of the Facet-Specific Growth Rates of BZM-I Crystals Grown at Different Flow Rates

flow rate (g h^–1^)	{011}-Side	{10*l̅*}-*b*-TE	{10*l̅*}-*a*-LE
5	3.7 ± 0.2	6.8	6.9 ± 0.3
35	3.2 ± 0.5	5.3 ± 0.5	9.5 ± 4.2

### 4.3 Growth Rates as a Function of Supersaturation for Pure BZM-I

**Figure 12:**
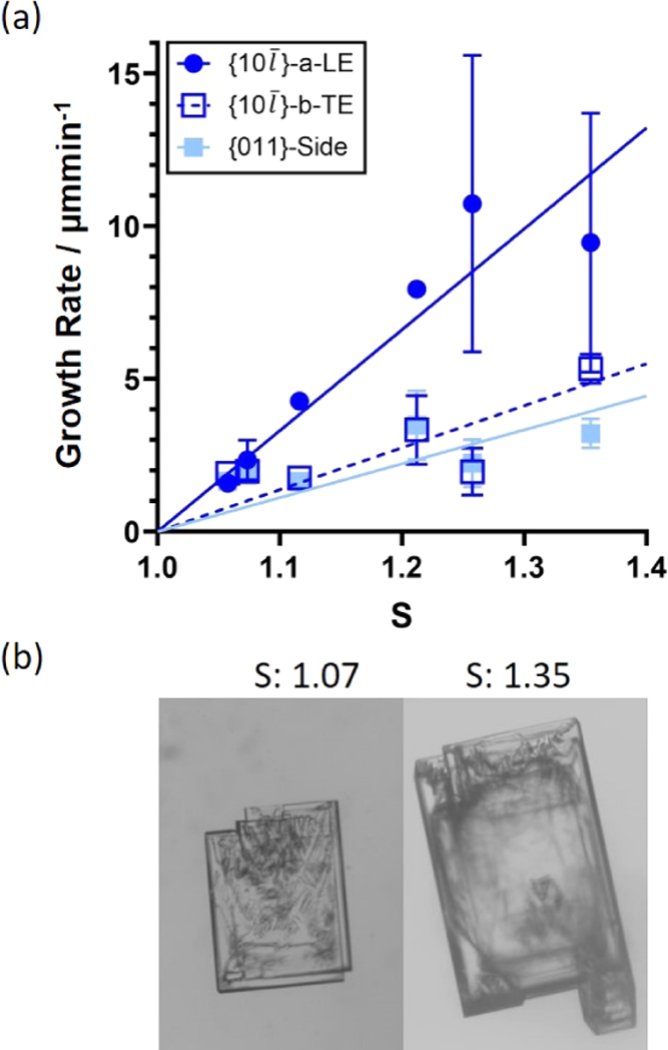
(a) Growth rates for pure BZM-I from IPA at 15 °C as a function of supersaturation at a constant flow rate of 35 g/h. All measurements were done in the flow cell. Where the face shows multiple layers, an average growth rate is given with the standard derivation as an error. Best linear fits to the data are added to aid the eye. (b) Images of grown crystals at two chosen supersaturations.

The derived growth rates of BZM-I pure from IPA at 15 °C in the flow cell are plotted against supersaturation in Figure 12. For those experiments where growth satellites on {10*l̅*} faces are seen, the average of the rate of all fronts was calculated with the standard deviation indicated. As seen in Figure 12 and in agreement with the above results, the {011} and {10*l̅*}-*b*-TE faces have very similar growth rates across all supersaturations, which is consistent with the typical plate-like morphologies. For the {10*l̅*}-*a*-LE, however, rates are significantly higher, and as the supersaturation increases, the generation of defects and observation of protuberance growth are significant (as seen in Figure 12, with larger error bars seen at supersaturations 1.25 and above, and larger protuberances on the LE facet the higher supersaturation). Growth rates of the {10*l̅*}-*a*-LE are approximately twice that of the other faces, which is in good accordance with the observed 1.9 aspect ratio of crystals grown by slow cooling.

### 4.4 BZM Growth Rates in the Presence of Additives Impacting the *L_a_* and *L_b_* Crystal Dimensions (*o*TAM and BA)

**Figure 13:**
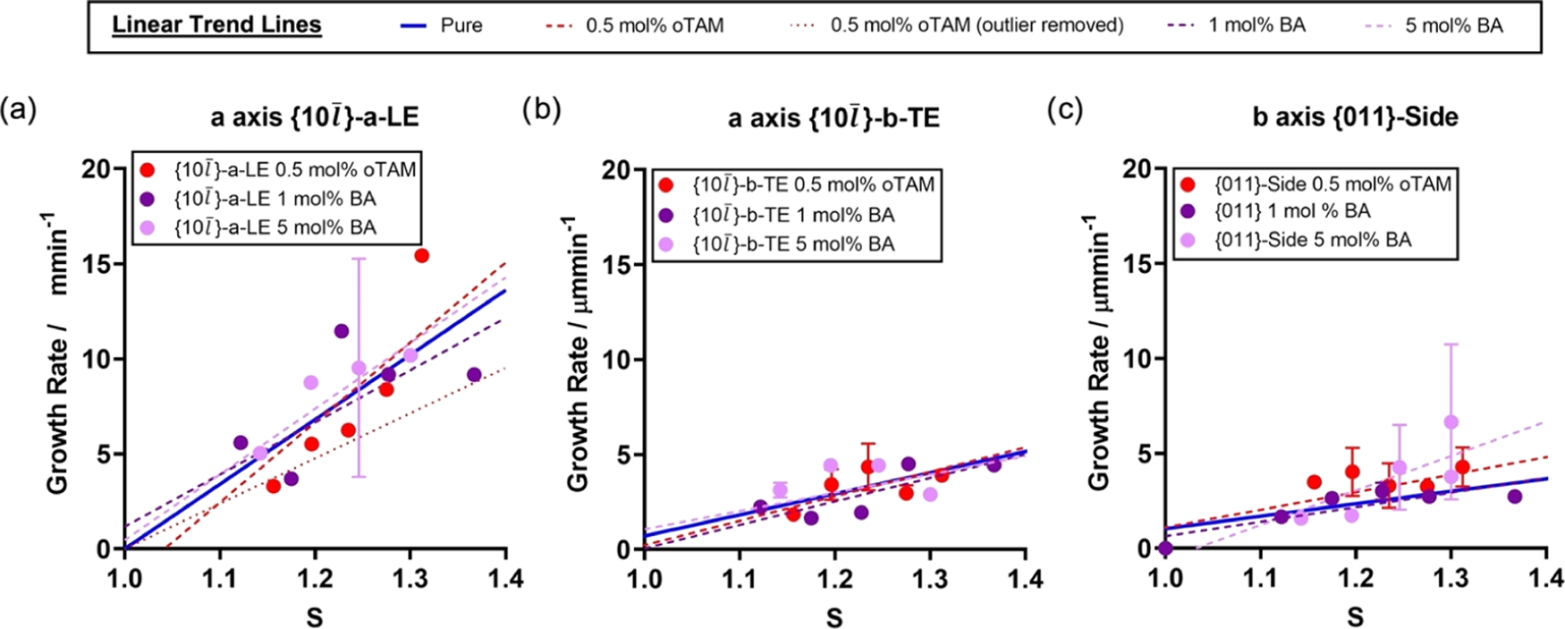
Growth rates of BZM-I grown from IPA at 15 °C pure and in the presence of 0.5 mol % of *o*TAM, 1 mol % BA, and 5 mol % BA. All measurements were done in the flow cell. Linear trends for growth in pure solution are shown in blue, while linear trends in the presence of the additives are shown as dashed lines.

Growth rates of BZM-I in the presence of BA and *o*TAM as a function of supersaturation are shown in Figure 13. As shown in Figure 5, crystals grown in the presence of 10 mol % BA and *o*TAM exhibit significant aspect ratio changes (*L_a_*/*L_b_*), moving from 1.9 for pure BZM-I crystals to 3.4 with BA and 0.2 with *o*TAM. While the use of a significant amount of additive (i.e., 10 mol %) is straightforward in a beaker solution crystallization, high additive concentrations result in unwanted nucleation events, which render the conditions unsuitable for the measurement of rates. For this reason, our rate measurements can only be done for lower additive concentrations, typically between 0.5 and 5 mol % depending on the impurity (SI).

Growth rates of BZM-I in the presence of BA and *o*TAM as a function of supersaturation are shown in Figure 13. As shown in Figure 5, the BA typically elongates the crystal morphologies along the *a*-axis while the *o*TAM reduces significantly the *a*-axis growth. *L_a_*/*L_b_* aspect ratios are 1.9 for pure BZM-I crystals, 3.4 for BZM-I crystals grown in the presence of 10 mol % of BA, and 0.2 for BZM-I crystals grown in the presence of 10 mol % of *o*TAM. While the use of a significant amount of additive (i.e., 10 mol %) is straightforward from a beaker solution crystallization, high additive concentrations result in unwanted nucleation events, which render the conditions unsuitable for the measurement of rates. For this reason, our rate measurements can only be done for lower additive concentrations, typically between 0.5 and 5 mol % depending on the impurity (SI).

Crystal growth rates of BZM-I in the presence of 1 and 5 mol % of BA as well as 0.5 mol % of *o*TAM are shown in Figure 13. First, the {10*l̅*}-*b*-TE data are discussed. Here, the additives have no impact on the {10*l̅*}-*b*-TE growth compared to pure solutions. Second, in the {10*l̅*}-*a*-LE data, it is evident that the additives have some impact on the growth, but the data overall are very dispersed, perhaps due to the uneven complex growth seen in this facet and orientation both with and without additives. In the case of 0.5 mol % *o*TAM (red circles), there appears to be a reduction of the rates overall at lower supersaturations, but above *S* = 1.3, the overall rate becomes larger. Finally, for the rates of the {011}-facets, again all trends are similar, but the additives appear to increase the rates slightly along the side dimensions of the BZM-I crystals.

Overall, the growth variability along the {10*l̅*}-*a*-LE, together with the fact that the growth measurements can only be performed under small concentrations of impurities, leads to only subtle changes in rate trends in the presence of additives.

### 4.5 Characterization of BZM-I Crystals Grown in the Presence of Additives Impacting the *L_c_* Crystal Dimension (*m*TAM and *p*TAM)

Growth rates of BZM-I crystals in the presence of *p*TAM and *m*TAM were only measured at low concentrations (0.5 mol %), again, due to unwanted nucleation at higher concentrations (SI). Both these additives at these low concentrations showed no significant impact on either the {10*l̅*} or {011} growth rates; therefore, they are not shown here for simplicity (SI). Interestingly, the presence of small amounts of *p*TAM resulted in the growth of many satellites, which caused many difficulties in deriving a true growth rate of the system. The major effect of both these additives, however, was found to be on the thickness growth (*c*-axis) with crystals grown in the presence of *p*TAM and *m*TAM being significantly thinner than in pure solutions.

**Figure 14:**
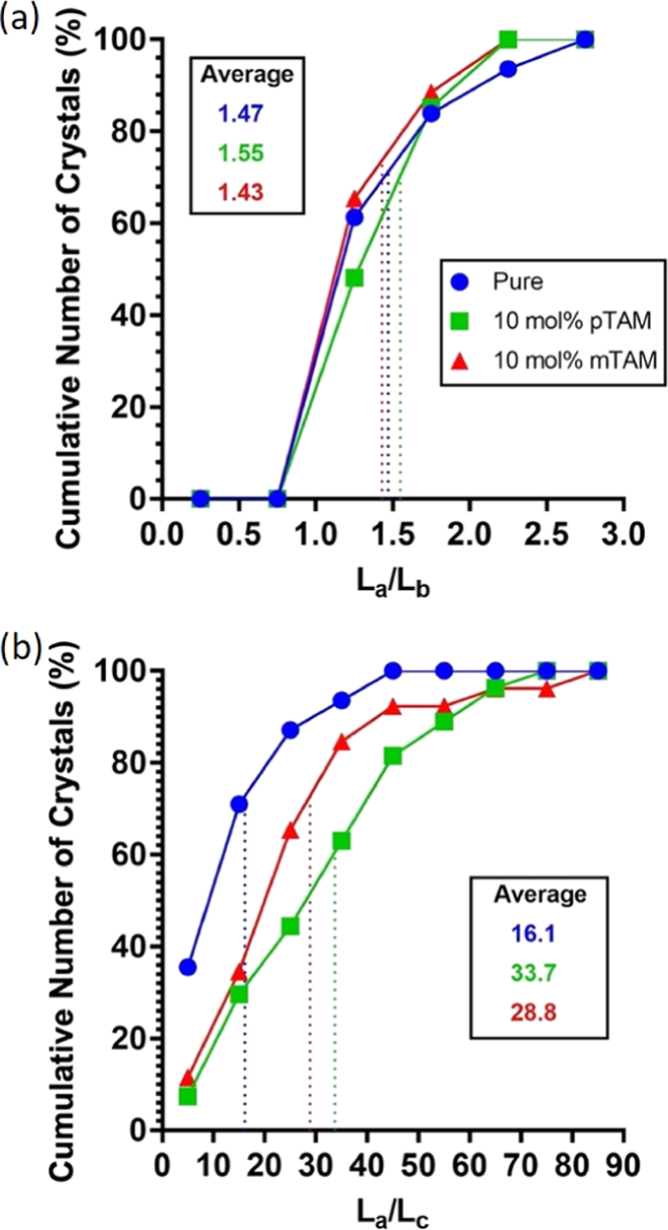
Cumulative distribution of aspect ratios for crystals of BZM-I grown from pure solution, 10 mol % *p*TAM, and 10 mol % *m*TAM under identical conditions for 24 h. Data are given as histograms using bin sizes of 0.5 (a) and 10 (b). *L_a_*, *L_b_*, and *L_c_* are the crystal dimensions along the *a*- and *b*-axes (usually length and width) and *c*-axis (thickness). The average *L_a_*/*L_c_* from these experiments is ∼1.5 or slightly smaller than for the crystals grown by slow cooling (∼1.9), differences which are ascribed to the crystallization conditions.

To quantify the effects of these additives on the thickness growth, crystals of BZM-I were grown in the presence of 0.5 and 10 mol % of these additives, and offline imaging was used to characterize all three crystal dimensions for a number of crystal populations (see Experimental Section). The data are shown in Figure 14 as cumulative distributions of *L_a_*/*L_c_* and *L_b_*/*L_c_* only for the 10 mol % experiments.

As expected, the *L_a_*/*L_b_* aspect ratio of the crystals is independent of the additive (Figure 14a). However, both additives lead to higher values of the aspect ratio *L_a_*/*L_c_* compared to pure solutions. The average value approximately doubles from 16 to ∼30 suggesting a halving of the crystal thickness under those conditions. This significant reduction in crystal thickness is a consequence of a decrease in the *c*-axis growth rate due to the additives. Further measurements were also done at 0.5 mol % *p*TAM and *m*TAM additive contents, results of which are shown in the SI. Here, an increase in the *L_a_*/*L_c_* aspect ratios for the experiments in the presence of the additives is also seen, however, to a much less extent than at 10 mol %. Overall, these results are consistent with the observed morphological changes.

One consequence of this thinning of the crystals by the additives is that it led to handling difficulties with the plates breaking more easily, especially at the moment of the sample dispersion on the sample plate. Hence, the means of the aspect ratio *L_a_*/*L_c_* of the crystals incorporated with impurities are expected to be higher before sample dispersion (since *L_a_* would be larger), with their distribution shifting further away from the pure BZM-I’s distribution to the right. Even, if this effect was kept to a minimum by handling the crystals as gently as possible, it still exists and cannot be quantified. The inhibiting effect of the impurities is clearly seen on the growth of the *c*-axis in these measurements. However, a comparison of the effect in-between the two impurities requires a softer sample dispersion as well as the measurement of more crystals to ensure the sample accurately represents the distributions.

## 5 Flow and Mass Transfer Simulations

**Figure 15:**
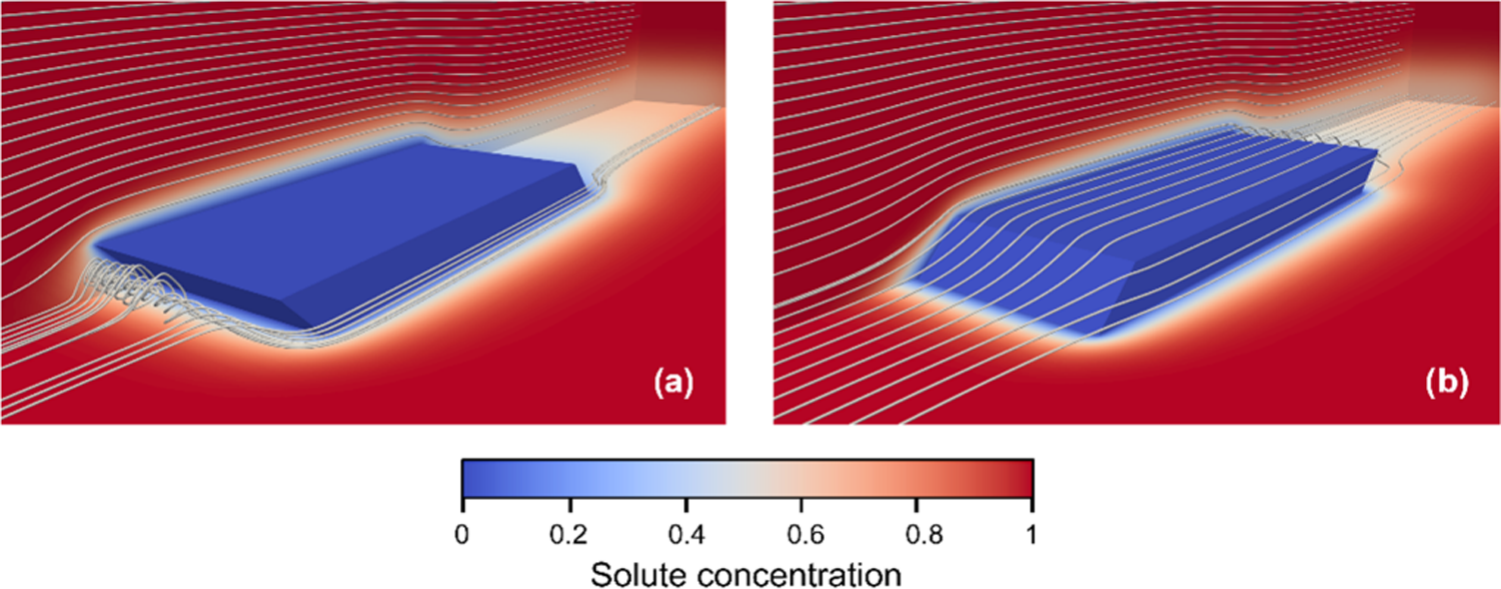
Contour maps of the solute concentration (nondimensional) and streamlines of the flow (white lines, flow direction from left to right) for the acute (a) and obtuse (b) angles of attack of the leading edge relative to the flow.

Computational fluid dynamics (CFD) simulations of the flow and solute mass transfer in the flow cell were performed for two different orientations of the {10*l̅*} facets of BZM-I crystals relative to the flow. Figure 15 shows the contour maps of the concentration and streamlines of the solution flow (white lines, flow direction from left to right) obtained from CFD for the (10*l̅*) and (1̅0*l*) geometries of facets (acute vs obtuse orientations) in the LE orientation. This is the solution flow attacking the {10*l̅*} facet at the LE with an acute or obtuse angle referred to as (10*l̅*) and (1̅0*l*), respectively, as per our naming convention. On the one hand, the simulations show the presence of a strong recirculation region in the upstream flow region when the crystal has the obtuse facet as leading edge (Figure 15a). On the other hand, any flow separation in the upstream flow region is observed when the crystal has the acute facet as the leading edge (Figure 15b). A global mass transfer rate for the entire crystal as well as local mass transfer rates for individual facets were calculated in both simulations.

**Figure 16:**
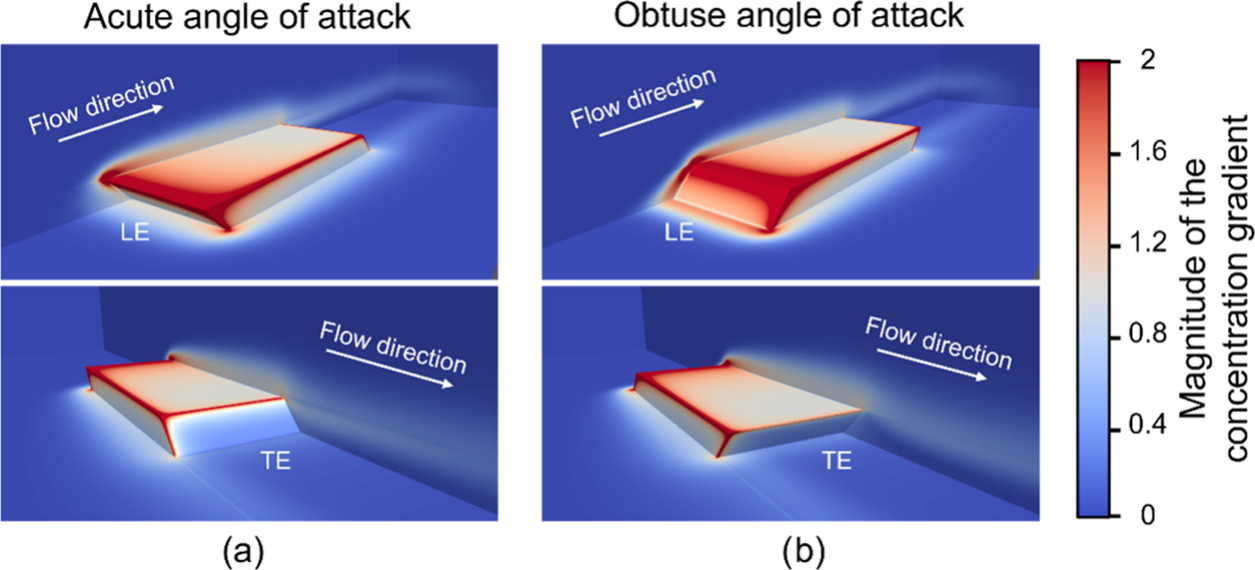
Contour maps of the magnitude of the gradient of solute concentration (nondimensional) for the acute (a) and obtuse (b) angles of attack of the leading edge relative to the flow. The two different views for each crystal orientation show the difference in the magnitude of mass transfer rates in the leading edges (LE) and tail edges (TE) of the crystal under flow conditions.

Despite the dissimilarities on the flow fields for the two crystal orientations, the CFD simulations indicate that the global mass transfer rate is only 0.4% higher for BZM-I with an acute LE than with an obtuse LE orientation. The CFD simulations also show similarities in the local mass transfer rates between these two crystal orientations but a stark difference between facets facing the flow (LE) or shielded from the flow (TE). For both acute and obtuse orientations, significantly higher gradients of concentration are seen at the LE than at the TE facets (Figure 16). Further to this, significantly higher mass transfer rates are localized at the edges of the crystal facets with the edges of the LE facet having the highest local mass transfer rates for both crystal orientations.

To summarize, CFD simulations have been carried out here to better understand mass transfer effects impacting the growth of BZM-I. These simulations are carried out by enforcing the boundary conditions that the rate of transfer of solute is much lower than the surface integration rate, which although not a reality, provides valuable insights into the growth effects arising from differences in mass transfer rates. Global and local mass transfers were found to be similar for both crystal orientations considered in the simulations (acute and obtuse at the LE) despite them having different flow fields. With regards to local mass transfer, LE facets were found to have significantly higher influx of BZM solute than TE facets, which is in good agreement with the experiments. The simulations also showed higher mass transfer rates at the edges of the facets rather than the center, which explains the appearance of satellites and the generation of hollows. Finally, there are no significant differences in mass transfer profiles between the acute and obtuse LE orientations studied here, which implies then the differences observed experimentally between these two orientations must be due to differences other than those generated by solution flow, such as growth dispersion or impurity profile effects.

## 6 Discussion

The crystal morphologies of BZM-I have been studied in detail as well as their growth both under static and flow conditions, as a function of orientation and in the presence of additives. All this work has revealed intimate details of the mechanism of growth of BZM-I and the consequences of such mechanisms.

Growth experiments of BZM-I have shown that while the {10*l̅*} facets are significantly impacted by solution flow, {011} facets are not. Growth of the {10*l̅*} facets is thus partially limited by mass transfer with rates dependent on both the solution velocity and the relative orientation of the facets with regards to the solution flow. When the {10*l̅*} facet occupies the LE position, a significant enhancement (two- to threefold) of the growth rate is observed in comparison to stagnant conditions. The CFD simulations confirm that the local mass transfer of solute is significantly higher in the LE than in the TE {10*l̅*} facets. Further to this, the simulations also show a higher local mass transfer at the edges of the LE {10*l̅*} facets than in the center. Such mass transfer limitations can mean that the rate of solute molecules arriving at the edges and corners will be higher than in the face centers, which results in the generation of hollows.^23−26^ Completion of new layers on top of the hollows results in inclusions, also common in BZM-I. While the phenomenon of hollow crystals is commonly observed in needles grown at high supersaturations and temperatures,^27,28^ it has been reported less often in plates and at much lower supersaturations, as is the case for BZM-I. Further to this, the variation of local supersaturation across the surface of the crystal will enhance the growth and surface nucleation rates of edges compared to the face centers and may lead to the formation of protuberance and satellites, also seen in this work. Optical and SEM images of the BZM-I crystals clearly show the common presence of hollows, inclusions, satellites, and protuberances on the {10*l̅*} facets, all a consequence of local mass transfer effects and the growth mechanism of such facets being diffusion controlled.

With regards to the impact of additives on the growth kinetics of BZM-I and the resulting morphologies, four different additives previously reported to alter the morphology of BZM-I have been studied here. Our experimental data have shown that these additives at low concentrations (>0–5 mol %) have almost no effect on the growth rates or morphologies of BZM-I, thus the additives cannot be considered “effective”. This behavior contrasts with many examples in the literature in which very low levels of additive (0.5–1 mol %) cause major changes in the rates; examples of effective growth inhibition with additives are *p*-aminobenzoic acid in the presence of 4-amino-3-methoxybenzoic acid or 4-amino-3-nitrobenzoic acid,^29^ urea grown in the presence of just 1 mol % of biuret,^30^ or benzophenone grown in the presence of just 3 mol % of 4-methylbenzophenone.^31^

**Figure 17:**
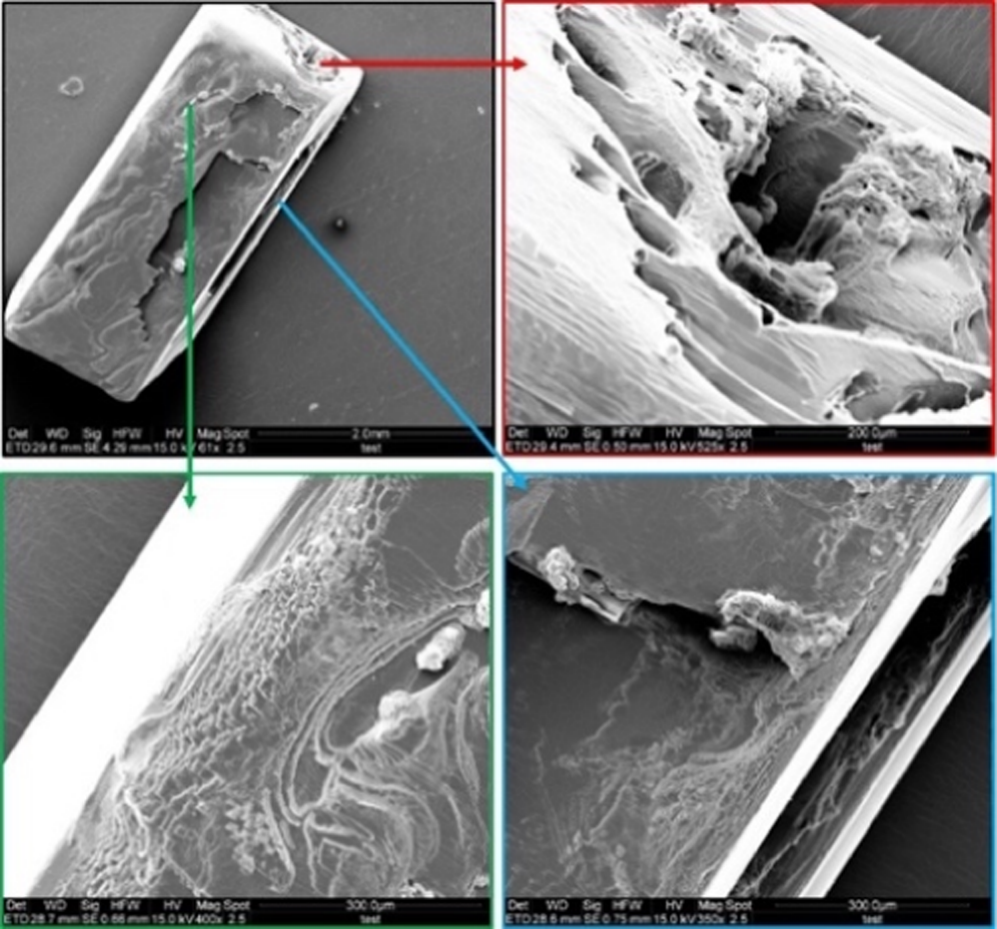
BZM-I crystal grown in the presence of additives shows hollows in all three facets.

One explanation as to why the BZM-I can continue growing even in the presence of additives may be because it grows in a very defected manner even when pure (as discussed above). Thus, additives may be removed from the interface either by incorporation in vacancies and inclusions or through the formation of solid solutions (Figure 17). A second explanation is that the additives can form solid solutions with BZM-I. Analysis of BZM-I powders grown in the presence of *m*TAM by pXRD revealed that BZM-I was the only phase present but also that some diffraction peaks shifted toward larger *d*-spacings. This result indicates that solid solutions are indeed forming between BZM-I and *m*TAM. This would explain the less dramatic impact of the additive on the growth rate of BZM-I.

**Figure 18:**
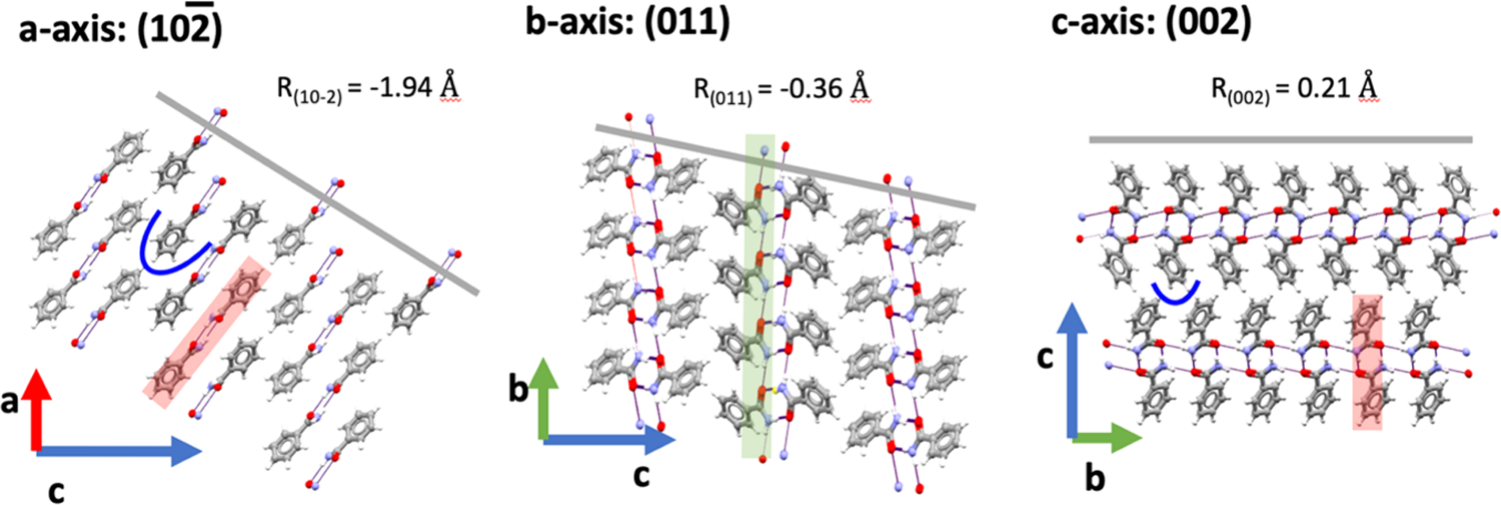
Structure and interactions along the three axes in BZM-I with facets depicted as gray planes. HB dimers in BZM are indicated in red, HB side chains in green, and key aromatic ring sites highlighted with a blue line. The rugosity of each of the crystal planes (*R* in Å) is given.^30,31^

Only when the additive concentrations become significant (∼10 mol %) is their impact on the morphology of BZM-I clearly seen. BZM-I grows involving either (i) HB dimers, (ii) HB side chains, or (iii) aromatic ring interactions. A molecular representation of the different facets is shown in Figure 18. Both the *a*-axis and the *c*-axis step growth involves aromatic ring attachment as well as HB dimers, while the *b*-axis step growth involves the side-HB chains. Since BA is the only additive that removes the possibility of side HBs, at 10 mol %, the *b*-axis growth becomes affected; thus, the crystals appear more elongated (Figure 5); however, the effect is relatively small given the high concentration of the additive. For the *o*TAM, *m*TAM, and *p*TAM, the modification of the ring through the addition of a methyl group at the ortho, meta, or para position will most likely impact either the *a*-axis or the *c*-axis growth since here, the ring-to-ring interactions are key for the step growth. Indeed, *o*TAM appears to significantly impact the a-axis growth while the *m*TAM and *p*TAM impact the *c*-axis growth at 10 mol %, as shown in our analysis of morphologies and aspect ratios. Predicting whether these additives will impact the a-axis over the *c*-axis growth more effectively is difficult, but the additives do seem to selectively impede the growth of one of the faces and not the other. This rationale has been discussed by Leiserowitz et al.^9^ in detail previously. Overall, while the previous findings on the additive impact on the crystal growth of BZM-I crystals have been reproduced at high additive concentrations, our detailed growth analyses also show that at low concentrations, the additives have hardly any effect on the growth of BZM-I.

**Figure 19:**
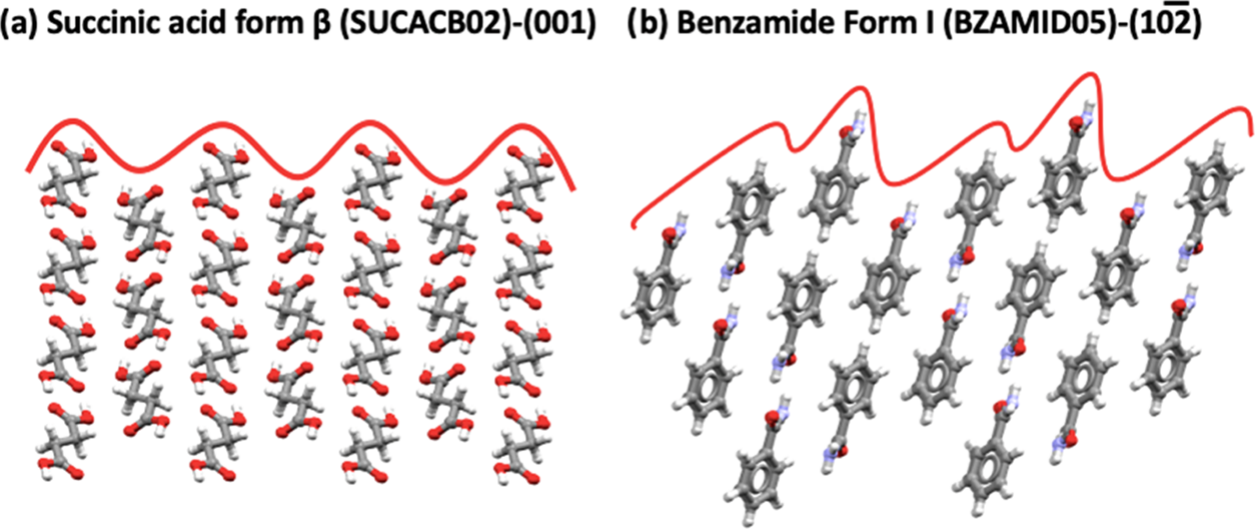
Structure of facets impacted by solution flow in succinic acid form β (a) and benzamide form I (b). A line has been added to help visualization of surface roughness.

Finally, given that mass transfer effects a major influence on the growth kinetics of the {10*l̅*} facets impacting rates, defect generation, and possibly impurity tolerance, the crystal structure of these facets at the molecular level was further explored here. The relevant surface of the BZM-I system -(102̅) facet- was considered together with the relevant surface in β succinic acid -(001) facet-, for which similar effects have been reported. In the case of β succinic acid, Mullin and Whiting also found that both the orientation as well as the solution flow rate impacted the (001) growth rates from aqueous solutions.^32^Figure 19 shows that both relevant facets affected by mass transfer rates are very rough in nature at the molecular level, displaying significant structural level rugosity and rougher structure than the other surfaces in the crystal, which display normal growth behavior.^33,34^ Because of such rugosity, it is possible that as soon as solute molecules are able to diffuse into the surface ridges, incorporation is very rapid, and thus the process becomes at least partially mass transfer controlled. Therefore, it is possible to make a preliminary link between structure and kinetics of growth controlled by mass transfer. Facets whose growth are limited by the transfer of solute to the surface, as we have seen here, will grow at different rates depending on their orientation relative to the solution flow.

## 7 Conclusions

Understanding the mechanisms of crystal growth at the molecular level and the impact of additives on them is key for crystallization development. Here, the crystal growth behavior of the historically important system BZM-I has been studied under flow conditions. The morphology of BZM-I can be dramatically altered when grown in the presence of certain facet-specific growth-inhibiting additives. Through the measurement of facet-specific crystal growth rates, the effect of additives on individual crystal facets can be examined.

In the case of BZM-I, a relativity large amount of the additives studied here (≈10 mol %) was needed for said morphology changes to be observed. At the additive levels used in growth rate experiments (0.5–5 mol %), additive effects are overshadowed by solution dynamics effects. BZM-I crystals grown under solution flow are of a reduced quality with increasing instances of features such as hollows, perturbations, and satellite crystals, with {10*l̅*} facets being most affected. While the growth rate of the {10*l̅*} facets is highly influenced by the solution flow, the growth rate of the {011} facet is not. This difference is attributed to differing growth mechanisms, with the {10*l̅*} facets showing evidence of diffusion-controlled growth. Computational fluid dynamics calculations show that there is a significantly higher mass transfer in the LE facets than the TE facet and that the edges and corners of the {10*l̅*}-LE facet have a higher concentration gradient than the center of the facet, which fits with our finding that this facet shows the most uneven growth in the FC.

Finally, an important link is made between a facet’s molecular rugosity and its growth rate being, as a consequence, controlled by mass transfer, thus being heavily affected by solution flow. The rough facet may preferentially grow through rough growth mechanisms, thus becoming highly sensitive to the available solute transfer rates. It is proposed that such rough growth may allow a facet to tolerate the effect of the additives better as the growth is already disrupted and additives get simply incorporated into the crystal via a whole range of defects.

## Supporting Information Available

Solubility data; PXRD data; image processing description; further growth rate data; characterization of crystal thickness; and computational fluid dynamics simulations details (PDF)
