# Supplementary material for: Complex Growth of Benzamide Form I: Effect of Additives, Solution Flow, and Surface Rugosity
Source: Cryst Growth Des. 2022 Sep 27;22(10):6248–61. doi: 10.1021/acs.cgd.2c00842 (PMC9542702; doi:10.1021/acs.cgd.2c00842)
Supplement: Supplementary file 1 — cg2c00842_si_001.pdf [file cg2c00842_si_001.pdf]

Supporting Information

Complex Growth of Benzamide Form I: Effect of Additives, Solution Flow and Surface Rugosity

Caroline A. Offiler[a], Claudio Pereira da Fonte[a], Weronika Kras[a], Petros Neoptolemou[a], Roger J. Davey[a], Thomas Vetter[b], Aurora J. Cruz-Cabeza[a*]

[a]Department of Chemical Engineering and Analytical Science, University of Manchester, Manchester M13 9PL, U.K.

[b]Department of Solid Form Science, H. Lundbeck A/S, Ottiliavej 9, 2500, Copenhagen (Denmark)

[*]Email: aurora.cruzcabeza@manchester.ac.uk

Contents

1. Solubility Tests 2

1.1 Method 2

1.2. DSC of equilibrated crystallites from solubility experiments 3

1.3. PXRD of equilibrated crystallites from solubility experiments 4

2. Preferred Orientation PXRD 6

3. Image Processing 6

4. Additional Growth Rate Data 7

5. Characterisation of BZM-I crystals grown in the presence of additives impacting the L_c crystal dimension (0.5 mol% mTAM and 0.5 mol% pTAM) 9

6. Computational Fluid Dynamics Calculations 10

7. References 12

1. Solubility Tests

1.1 Method

Slurries containing an excess of benzamide solids in IPA were prepared in sealed glass vials using the appropriate amounts of additives. The vials were held at 15°C in a temperature-controlled device (Polar Bear Plus Crystal, Cambridge Reactor Design) for 48 h under constant stirring (250 rpm, magnetic stirrer). 5 x 0.5 ml of saturated solutions were then retrieved with the aid of a syringe, filtered (0.45 µm filter) and added to five independent vials. The mass of the vials and solutions was recorded using an analytical balance (Fisher scientific, ±0.0001g). The samples were then left to evaporate in a vacuum oven (40°C) for over 24 hours. After all solvent had evaporated, the masses of the residual solids were recorded, and the average solubility calculated from the five independent measurements. For the samples containing additives, five independent sets of slurries were prepared, each containing a different BZM: additive ratio. Since the amounts of additives used were small (between 0.1 to 10 mol% on total amount of BM in the slurry), it was assumed that the additives fully dissolved in the saturated solutions from this the theoretical mol% of additive w.r.t the mols of BNZ in the saturated solution is calculated. This was confirmed experimentally by analysing the excess solids from the slurries (DSC and pXRD) which corresponded to pure BZM form I in all cases except at high levels of pTAM (over 18 mol %) and oTAM (over 10 mol%) (ESI). Knowing the original amount of additive the ratio of additive to BZM was then calculated after obtaining the total mass of solids from the equilibrated solution after the solvent had evaporated. For each additive a solubility curve is plotted and trend line fitted (Figure ESI-1), from this the solubility at the desired mol % additives calculated.

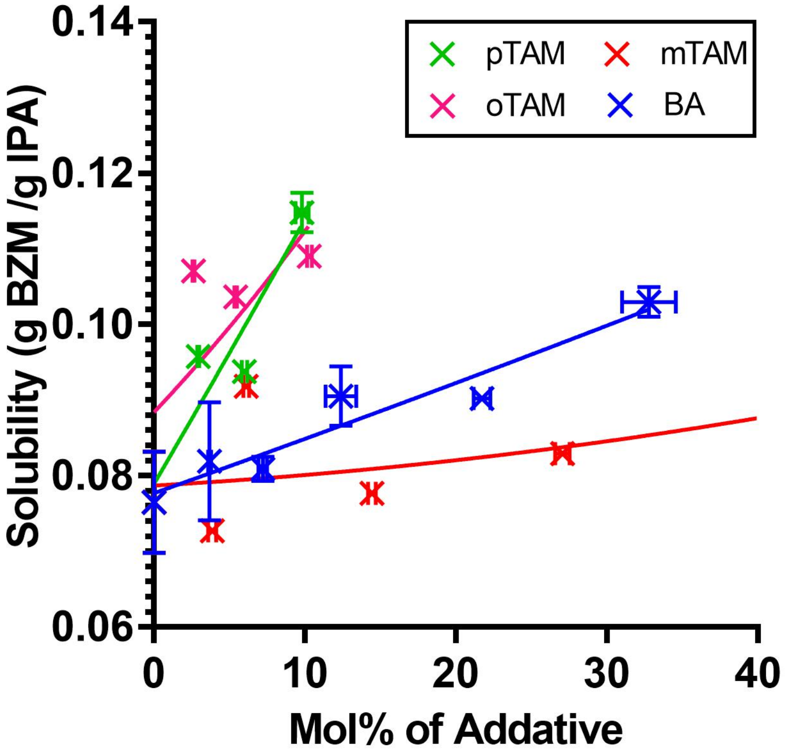

Figure ESI-1. Effect of the additives: oTAM, pTAM, mTAM and BA on the solubility of BZM-I in IPA at 15°C.

1.2. DSC of equilibrated crystallites from solubility experiments

Figure ESI-2 shows the DSC spectra preformed on the residual solids obtained in solubility studies. Note that the wt% of additive is based on the amount of benzamide and additive placed in the vessel at the start of the experiment and is not equal to the wt% of additive in the saturated solution. With oTAM and pTAM additional phases are seen in the DCS spectra at additive levels over 10 wt% however in the case of both additives this corresponds to an additive mol% in saturated solution >40 far above what was used in this work. It is possible that co-crystals form in this region.

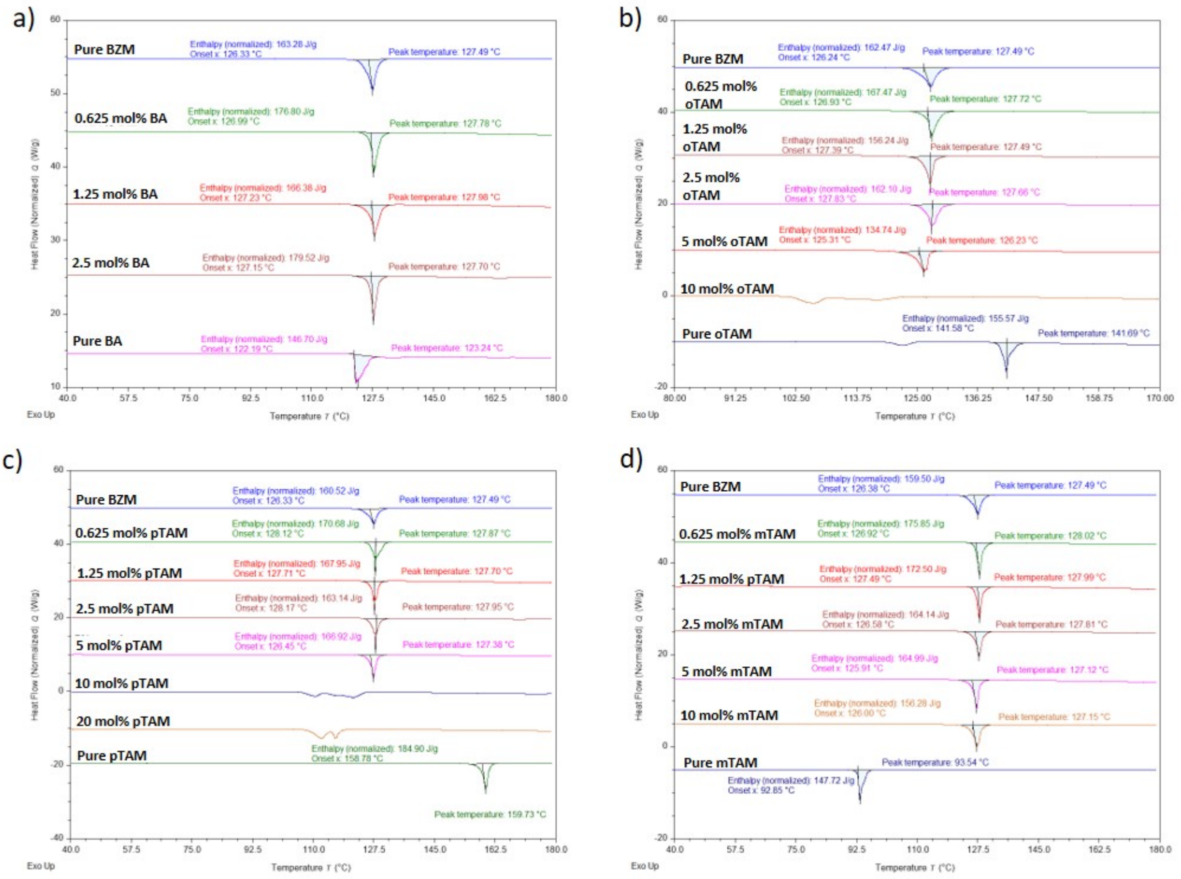

Figure ESI-2. DSC spectra of the residual solids from solubility tests of BZM in the presence of a) BA, b) oTAM, c) pTAM and d) mTAM. The amount of additive is relative to the total mass of benzamide used in the solubility test.

1.3. PXRD of equilibrated crystallites from solubility experiments

Figure ESI-3 shows the PXRD spectra preformed on the residual solids obtained in solubility studies. In the additives concentration used in this work (≤10 mol%) no peaks other than those corresponding to BZM-I are seen. Note in cases where addition peaks are seen in the PXRD spectra (>10 mol % oTAM and >18 mol% pTAM wrt BZM in the saturated solution) the corresponding slurries were not considered for solubility measurements. As seen in Figure ESI-3d in the presence of mTAM some BZM-I peaks are shifted to higher 2θ values evident of solid solution formation.

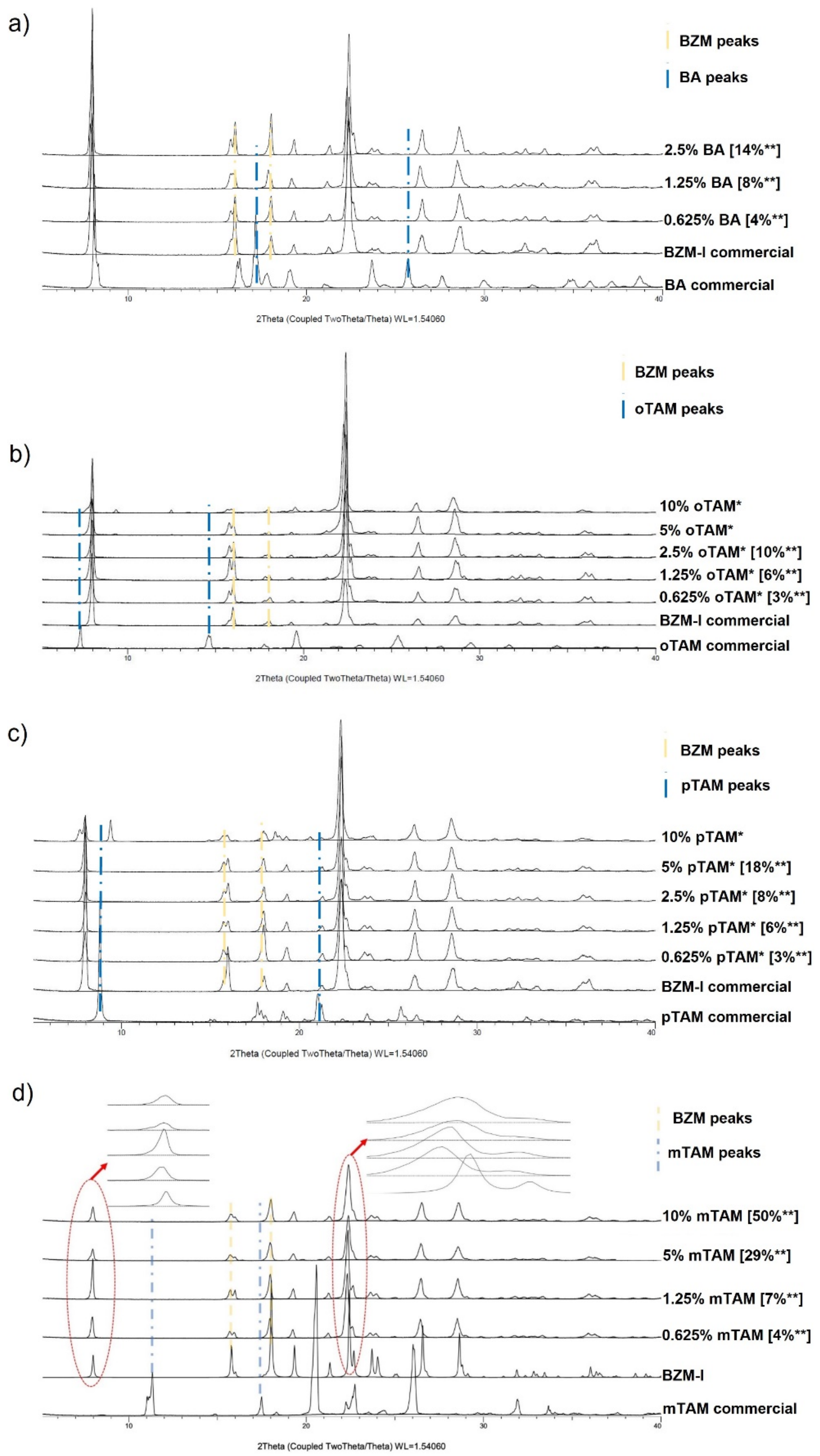

Figure ESI-3. Diffractograms of crystallites obtained from slurries containing BZM and the following additives: a) BA, b) oTAM, c) pTAM and d) mTAM. Percentage contents of additive indicated by single asterisk refer to the initial wt.% of additive added to the slurries wrt to total amount of additive and BZM added, while double asterisks indicate mol% wrt to BZM mol in saturated solutions if negligible amounts incorporated in the slurry crystallites.

2. Preferred Orientation PXRD

Preferred orientation experiments are carried out on BZM-I crystals grown by slow evaporation in IPA and water (80:20 wt %). Firstly the crystal is lay flat on the sample holder displaying the dominate face (Figure ESI-4 - Front face) the expected peak for the {002} facet is seen. To see the side facets a knife is used to slice of the sides of the crystal this is placed in the sample holder sat on its sliced edge (Figure ESI-4- side faces). Peaks corresponding to the {011} and $\{10\bar{l}\}$ facets are seen. Our data suggests the index of the $\{10\bar{l}\}$ facet is 4.

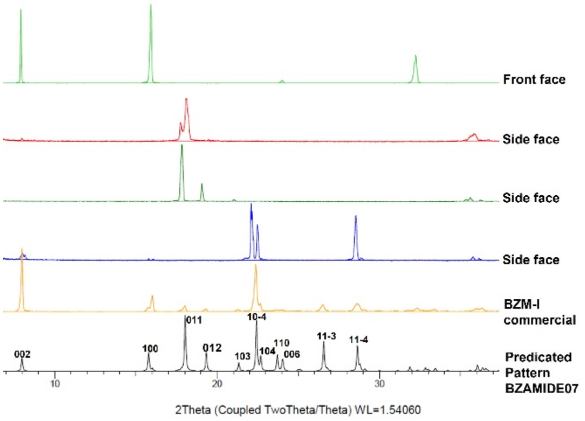

Figure ESI-4. Preferential orientation face indexing preformed on BZM crystals grown by slow evaporation in a mixture of water and IPA.

3. Image Processing

In this work we encounter crystals that show uneven growth across a facet resulting in perturbations and satellite crystals growing from the bulk crystal. In these cases the, convexhull, shape closing method used in our code (detailed in previous work[1]) will not provide an accurate representation of the crystal shape (Figure ESI-5). A different method for isolating the crystal shape must be used, Figure ESI-5 shows the original image and the results of closing the shape using three different methods. Two approaches can be used to do this: a morphological closing can be used in which a shape of set size is used to outline and close the shape. Alternatively, we can avoid the shape closing step by adjusting the binarization so that the crystals full outline is visible in the binary image, filling all shapes in the image (the crystal and noise) the crystal shape is picked out based on its area and solidity (area/convex hull area). This method is used in preference as it

does not alter the shape outline. However to be used the complete crystal outline must be distinguishable from the background and in some cases this is not possible this method also required the threshold used for binarization to be optimised prior to analysis.

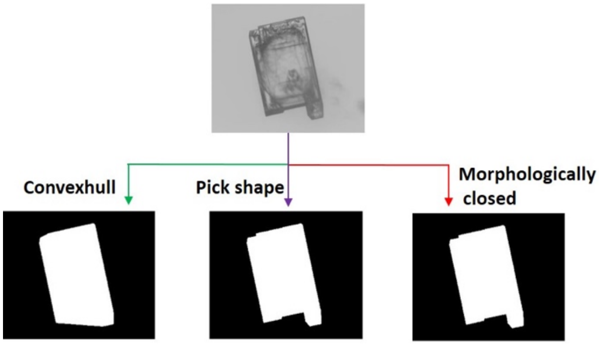

Figure ESI-5. Example of three different methods for obtaining the crystal shape: the convexhull method, picking the shape based on its area and solidity and using a morphological structuring element to close the shape.

4. Additional Growth Rate Data

Growth rate data collected for BZM-I grown in the presence of 0.5 mol% pTAM and mTAM is shown below. Growth rate measurements were performed in the presence of 0.5 mol% toluamide due to nucleation on the crystal surface seen at higher doping levels most prevalent for pTAM. Figure ESI-6 shows a BZM-I crystal grown in the flow cell in the presence of 1 mol% pTAM due to the number of satellite crystals present it is not possible to obtain facet growth rate for such crystals and so lower additive levels were required. For comparison between the toluamide additives all growth experiments were performed at 0.5 mol%.

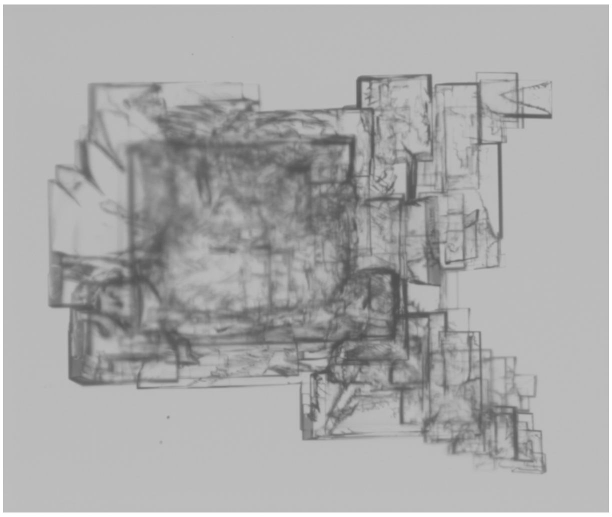

Figure ESI-6. Crystal grown in for 45 min in the FC in the presence of 1 mol % pTAM. (S=1.26, 15°C and flow rate 35g/h).

In the case form mTAM growth rates are within the error of those obtained for pure BZM-I. However, crystals grown in the presence of mTAM show a reduction in the perturbations and satellite crystals present. In contrast pTAM even at 0.5 mol% leads to a noticeable increase in satellite crystals. This explains the slightly higher growth rates seen for the $\{10\bar{l}\}$ facet in the presence of pTAM.

Error bars are seen in pTAM growth rate data while with mTAM growth rate data flows a much smother trend, these observations agree with the slow cooling data.

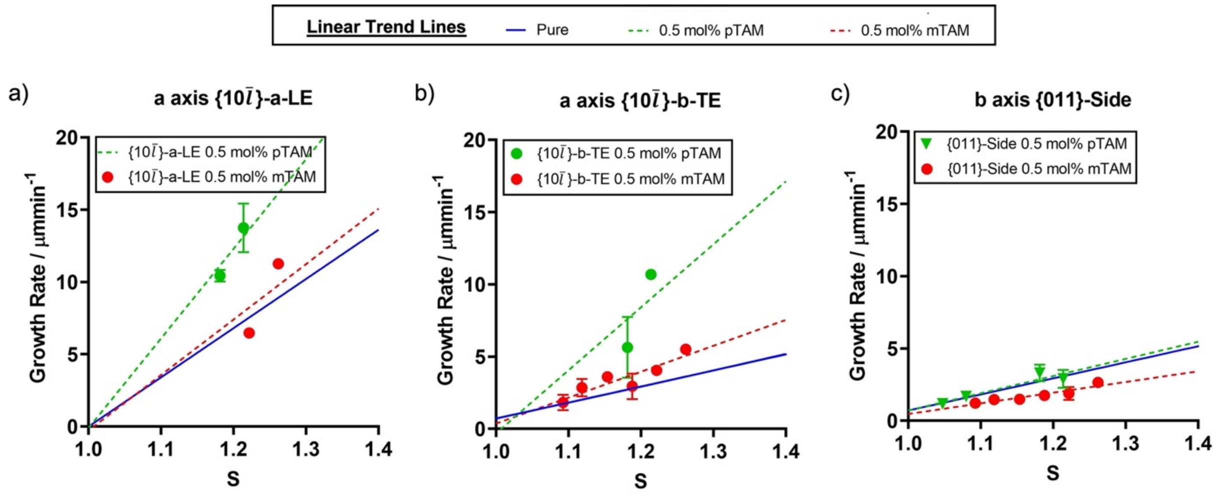

Figure ESI-7. Growth rates of BZM-I grown from IPA 15C in pure and in the presence of 0.5 mol% pTAM and 0.5 mol% mTAM. The linear trends for growth in pure solution are shown in blue whilst linear trends in the presence of additives are shown as dashed lines. Note some crystals were not perfectly oriented this is not expected to effect the $\{011\}$ facet growth rate and have a limited effect on the $\{10\bar{l}\}$-TE and so data points are included for these facets but not the $\{10\bar{l}\}$.

5. Characterisation of BZM-I crystals grown in the presence of additives impacting the L_c crystal dimension (0.5 mol% mTAM and 0.5 mol% pTAM)

Distribution of the aspect ratios L_a/L_b and L_a/L_c for crystals of BZM-I grown from pure solution, 0.5 mol% pTAM and 0.5 mol% mTAM are shown in Figure ESI-8.[2] An increase in the L_a/L_c aspect ratio is observed in the presence of 0.5 mol% pTAM although to a much lesser extent that in the presence of 10 mol% additive. This can be attributed to a reduction in Lc growth as the average La/Lb aspect ratio remains similar to that of the pure crystals. With 0.5 mol% mTAM no change significant change in the crystal dimensions are seen.

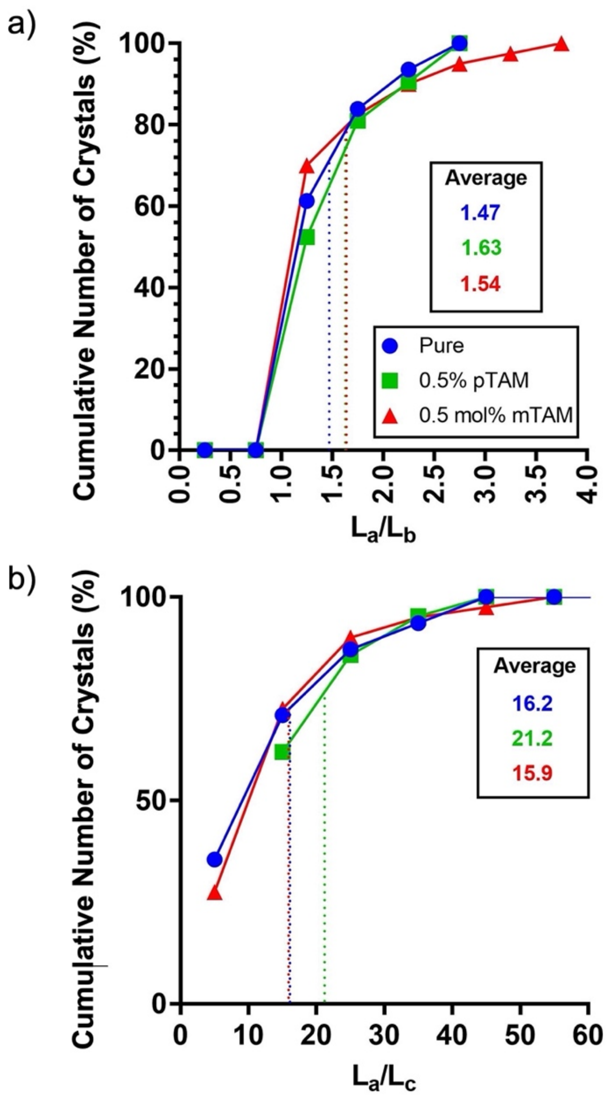

Figure ESI-8. Cumulative distribution of aspect ratios for crystals of BZM-I grown from pure solution, 0.5 mol% pTAM and 0.5 mol% mTAM under identical conditions for 24 h. Data is given as histograms using bin sizes of 0.5 (a) and 10 (b). La, L_b and Lc are the crystal dimensions along the a and b axis (usually length and width) and c axis (thickness).

6. Computational Fluid Dynamics Calculations

The flow field and concentration distribution inside the flow cell were obtained by solving numerically the Navier-Stokes equations for a fluid of density ρ and viscosity μ,

$$\nabla \cdot \mathbf{u} = 0$$

$$Re(\mathbf{u} \cdot \nabla\mathbf{u}) = -\nabla p + \nabla^2\mathbf{u}$$

and the equation that describes the diffusive and advective transport of the concentration, c, of benzamide in the flow,

$$Pe(\mathbf{u} \cdot \nabla c) = \nabla^2 c$$

where $\mathbf{u}$ is the velocity field normalised by the inlet velocity U, and p is the gauge pressure field normalised by the characteristic pressure $p_0 = \mu U/h$. The variable h is a characteristic dimension of the flow, taken to be in this work the height of the BZM-I crystal. The concentration c is normalised by the inlet concentration of benzamide in the solution, c_0. The normalisation of the equation variables leads to the appearance of two dimensionless groups: the Reynolds number $Re = \rho hU/\mu$; and the Péclet number $Pe = hU/D$, where D is the molecular diffusivity of benzamide in the solvent.

The flow domain consists of a prismatic box of height $H/h = 6.3$, width $W/h = 11.25$ and length $L/h = 43.2$. At the bottom of the domain, a $44.45°$ right rhombic prism crystal geometry with fixed size and aspect ratio $w/h = 9$ (width/height) and $L/h = 10.8$ (length/height) was placed. We performed simulations for two different crystal orientations corresponding to the crystal front face making obtuse or acute angles of attack with regards to the sample holder plane in the direction of the flow. Table ESI-1 summarises the boundary conditions adopted in the CFD model.

Table ESI-1. Boundary conditions of the CFD model

Boundary	Flow	Mass transport
Sample holder plane	No-slip	Zero diffusive flux
Crystal surfaces	No-slip	$c = 0$
Inlet	$U = 1$ normal to the surface	$c = 1$
Outlet	$p = 0$	Zero diffusive flux
Remaining surfaces	Symmetry	Symmetry

The flow domain was discretized with 4.7 thousand quadrilateral finite volume elements with variable size. The grid was refined close to the walls of the crystal, where higher gradients of

concentration were expected. Mesh independence tests showed that the adopted mesh was sufficiently refined to resolve both the flow and concentration fields.

The governing equations for the flow and mass transport were numerically solved with the open-source Finite Volume CFD code OpenFOAM v9.[3] Steady-state solutions of the flow and concentration fields were obtained with the solver *simpleFoam* using second-order interpolation schemes for the advective terms of the governing equations. The SIMPLE method was used to solve iteratively the velocity and pressure fields. The simulations were considered converged when the residuals of all the equations reached values smaller than 10^{-6} during the iterative solution process.

7. References

(1) Offiler, C. A.; Cruz-Cabeza, A. J.; Davey, R. J.; Vetter, T. Crystal Growth Cell Incorporating Automated Image Analysis Enabling Measurement of Facet Specific Crystal Growth Rates. Cryst. Growth Des. 2021, No. 22, 2837–2848, DIO:10.1021/acs.cgd.1c01019.

(2) Neoptolemou, P.; Goyal, N.; Cruz-Cabeza, A. J.; Kiss, A. A.; Milne, D. J.; Vetter, T. A Novel Image Analysis Technique for 2D Characterization of Overlapping Needle-like Crystals. Powder Technol. 2021, 116827, DIO:https://doi.org/10.1016/j.powtec.2021.09.017.

(3) OpenFOAM. https://openfoam.org/ (accessed 2019-02-21).
